# A Systems Approach to Interrogate Gene Expression Patterns in African American Men Presenting with Clinically Localized Prostate Cancer

**DOI:** 10.3390/cancers13205143

**Published:** 2021-10-14

**Authors:** Gary Hardiman, Stephen J. Savage, E. Starr Hazard, Willian A. da Silveira, Rebecca Morgan, Adam Harris, Melanie S. Jefferson, Robert C. Wilson, Susan Caulder, Linda Ambrose, Lewis Frey, Bethany Wolf, Sebastiano Gattoni-Celli, Chanita Hughes Halbert

**Affiliations:** 1 Department of Medicine, Medical University of South Carolina (MUSC), Charleston, SC 29425, USA; 2 Faculty of Medicine, Health and Life Sciences, School of Biological Sciences and Institute for Global Food Security, Queens University Belfast, Stranmillis Road, Belfast BT9 5AG, UK; willian.dasilveira@staffs.ac.uk (W.A.d.S.); rmorgan21@qub.ac.uk (R.M.); adam.harris@qub.ac.uk (A.H.); 3 Department of Urology, Medical University of South Carolina (MUSC), Charleston, SC 29425, USA; savages@musc.edu; 4 Ralph H. Johnson VA Medical Center, Charleston, SC 29401, USA; caulder@musc.edu (S.C.); ambrosel@musc.edu (L.A.); Sebastiano.Gattoni-Celli@bms.com (S.G.-C.); 5 Department of Radiation Oncology, Medical University of South Carolina (MUSC), Charleston, SC 29425, USA; 6 Academic Affairs Faculty, Medical University of South Carolina (MUSC), Charleston, SC 29425, USA; hazardes3@gmail.com; 7 Hollings Cancer Center, Medical University of South Carolina (MUSC), Charleston, SC 29425, USA; sweatma@musc.edu; 8 Department of Pathology and Laboratory Medicine, Medical University of South Carolina (MUSC), Charleston, SC 29425, USA; relaxingbob@gmail.com; 9 Biomedical Informatics Center (BMIC), Medical University of South Carolina (MUSC), Charleston, SC 29425, USA; frey@musc.edu; 10 Department of Public Health Sciences, Medical University of South Carolina (MUSC), Charleston, SC 29425, USA; wolfb@musc.edu; 11 Department of Psychiatry & Behavioral Sciences, Medical University of South Carolina (MUSC), Charleston, SC 29425, USA; 12 Department of Population and Public Health Sciences, University of Southern California, Los Angeles, CA 90032, USA; 13 Norris Comprehensive Cancer Center, University of Southern California, Los Angeles, CA 90033, USA

**Keywords:** stress, precision medicine, RNA-Seq, prostate, health disparities, African American, vitamin D, transcriptomics, health disparities

## Abstract

**Simple Summary:**

Men of African origin have a 2–3 times greater chance of developing prostate cancer than those of European origin, and of patients that are diagnosed with the disease, men of African descent are 2 times more likely to die compared to white men. Men of African origin are still greatly underrepresented in genetic studies and clinical trials. This, unfortunately, means that new discoveries in cancer treatment are missing key information on the group with a greater chance of mortality. The objective of this study was to increase our knowledge of prostate cancer in men undergoing a prostate biopsy. We carried out RNA sequencing of biopsy specimens and examined racial differences in prostate gene expression. A gene expression signature was uncovered which separated the men based on their race. Furthermore, within men of African descent this signature separated men with the most severe clinical characteristics.

**Abstract:**

An emerging theory about racial differences in cancer risk and outcomes is that psychological and social stressors influence cellular stress responses; however, limited empirical data are available on racial differences in cellular stress responses among men who are at risk for adverse prostate cancer outcomes. In this study, we undertook a systems approach to examine molecular profiles and cellular stress responses in an important segment of African American (AA) and European American (EA) men: men undergoing prostate biopsy. We assessed the prostate transcriptome with a single biopsy core via high throughput RNA sequencing (RNA-Seq). Transcriptomic analyses uncovered impacted biological pathways including PI3K-Akt signaling pathway, Neuroactive ligand-receptor interaction pathway, and ECM-receptor interaction. Additionally, 187 genes mapping to the Gene Ontology (GO) terms RNA binding, structural constituent of ribosome, SRP-dependent co-translational protein targeting to membrane and the biological pathways, translation, L13a-mediated translational silencing of Ceruloplasmin expression were differentially expressed (DE) between EA and AA. This signature allowed separation of AA and EA patients, and AA patients with the most severe clinical characteristics. AA patients with elevated expression levels of this genomic signature presented with higher Gleason scores, a greater number of positive core biopsies, elevated dehydroepiandrosterone sulfate levels and serum vitamin D deficiency. Protein-protein interaction (PPI) network analysis revealed a high degree of connectivity between these 187 proteins.

## 1. Introduction

Stress occurs when a person cannot cope efficiently with the daily physical or psychological demands placed on the body [1,2,3,4]. The primary hormonal stress mediators, catecholamines and glucocorticoids, elicit both beneficial and harmful effects. In the short term, they are required for allostasis, adaptation, cellular homeostasis, and survival. However, over an extended period, this leads to ‘allostatic load’ first defined by McEwen and Stellar [5] that can accelerate disease processes including cancer [6]. Allostatic load indicates disruption of biological processes in response to psychological and social stress. It is used as a marker of how much psychological and social stressors impact biological functioning [7,8,9]. Allostatic load values reflect chronic, steady state levels of stress as well as failure to terminate responses to acute stressors [10,11]. 

According to the National Center for Health Statistics (2013), men from racial minority groups continue to experience poor health outcomes compared to non-minority men. For instance, the life expectancy for African American (AA) men is significantly lower compared to the expectancy for white men. Precision medicine can play a significant role in reducing racial disparities in morbidity and mortality among minority men because these efforts are designed to individualize health care based on biological, behavioral, and social factors that contribute to disease risks and enhance health outcomes. However, the development and implementation of approaches for precision medicine is still limited among minority men because empirical data are lacking on the ways in which risk factors and protective variables work in this underserved population.

From a global perspective prostate cancer is among the most frequently diagnosed malignancies among men. In the United States it is the second leading cause of cancer-related mortality. Although its incidence has declined in recent years, overall prostate cancer-related mortality among AA men has increased [12,13,14]. Epidemiological studies suggest that AA men have greater prostate cancer incidence, morbidity, and mortality of compared to European-Americans (EA) [15]. The origin and causes of racial disparities in prostate cancer risk and outcomes remain to be fully elucidated. However, it is well established that socioeconomic and behavioral factors both contribute to disease progression. Distrust in the health care system and fear associated with detection and treatment have also been listed as reasons for late diagnosis in AA men [14]. Reduced access to health care services is a contributor, but even when access to health care systems is comparable in EA and AA men such as the Veterans Administration (VA), AA exhibit elevated serum PSA values and higher-grade tumors than EA even when the identical disease stage is considered [16,17]. Thus, access to medical care is essential but not adequate for eradicating racial disparities in prostate cancer outcomes.

Racial differences are common among cancers, examples include multiple myeloma, colorectal and breast cancer. Multiple myeloma is the most common hematologic cancer in patients of African descent [18]. Although it is a rare disease, diagnoses are twice as high in patients of African descent compared to European descent. Reasons for these differences are currently unknown and the disease remains uncurable [19,20,21]. In the USA, colorectal cancer patients of African descent experience decreased stage specific survival in comparison to patients of European descent irrespective of socioeconomical background, age, treatment, and stage at diagnosis [22]. Invasive breast cancer mortality rates also differ between patients of African and Northern European descent. In the USA, African American women are more likely to die from the disease than European-American women [23]. African American women also experience worse prognosis and decreased survival times compared to European-American women regardless of their socioeconomical background, age, and stage at diagnosis [24].

Prostate cancer (PC) has one of the greatest racial disparities among cancer types. In the U.S.A., African Americans have among the highest mortality and lowest survival rates of any race or ethnic group [25]. PC is also the top cause of cancer mortality among men of African descent living in Caribbean and Sub-Saharan Africa [26]. Similar disparities exist in the UK, where men of African descent are 2–3 times more likely to develop PC than white men and have a 30% higher mortality rate [27]. Non biological factors contributing to PC racial disparities include diminished societal trust in the medical community, level of education attained, and financial concerns. Access to healthcare plays an important role in PC mortality. However, African American men have higher PSA values and higher tumor grades even at the same cancer stage, compared with non-African American men within the same healthcare system such as the Veterans Administration in the USA [28]. The reasons underlying these racial differences remain an area of active research. Many biological explanations have been considered including dietary factors such as alcohol and nicotine intake, vitamin D deficiency and lycopene and isoflavone consumption [28,29,30,31,32]. African Americans have higher rates of variations in tumor suppressor genes such as *EPHB2* and are susceptible to higher prevalence of chromosome 8q24 variants which are linked with prostate cancer [33,34,35,36,37,38].

Racial differences in prostate cancer outcomes reflect racial variations in vitamin D levels [39]. Several studies have shown that approximately 60% of AA men have sub-optimal levels of circulating 25(OH)D [40,41]. Our research team previously demonstrated that supplementation with vitamin D3 eradicates racial differences in serum levels of 25(OH)D [42,43,44]. We previously carried out a systems level analysis of the prostate transcriptome in a cohort of AA men and EA men who had undergone a prostatectomy, following a diagnosis of localized prostate cancer. [28]. In this sample of 27 subjects (10 AA and 17 EA) who were randomized to vitamin D3 supplementation or placebo prior to surgical treatment, AA men exhibited increased expression of immune response and inflammatory related genes, suggesting that inflammation may contribute to disease development in AA. These inflammatory transcripts were modulated with vitamin D3 supplementation. This initial study revealed the existence of fundamental biological differences within the prostate between AA and EA men with prostate cancer.

In the absence of androgen receptor (AR) signaling, the glucocorticoid receptor (GR) is a major contributor to disease progression and drug resistance in prostate cancer. The consequences of glucocorticoid-activated GR signaling mediating protein expression related to tumor progression and drug resistance could influence patients with high endogenous cortisol levels or a susceptibility to GR signaling such as AA patients. A recent study based on AA patient derived cell lines indicated that glucocorticoid signaling is an important aspect of prostate cancer health disparities [45].

In this study, we examined molecular profiles and cellular stress responses in an important segment of AA and EA men: men undergoing prostate biopsy. The primary goal in the present study was to demonstrate the ability to assess the prostate transcriptome with a single biopsy core in a new patient cohort presenting with or at risk for early-stage prostate cancer. This information could guide precision medicine approaches tailored specifically for AA men. High throughput RNA sequencing (RNA-Seq) was performed, and analyses compared across racial groups. An additional goal of the present study was to correlate relevant health parameters of patients undergoing a core biopsy with the molecular profiles obtained from prostate tissue samples. This objective was consistent with increasing evidence that pro-inflammatory mechanisms and the hypothalamic pituitary adrenal axis are key mediators of the physiological stress or allostatic load, and previous work from this group which highlighted a molecular signature of inflammation in the AA prostate of men who have undergone a prostatectomy [28]. This study was approved by the Institutional Review Board at the Ralph H. Johnson Veteran’s Affairs Medical Center.

## 2. Results

### 2.1. Patient Cohort

We enrolled 33 AA and 27 EA men, 60 subjects in total, who had undergone a prostate biopsy. Table 1 provides the characteristics of the subjects, distributions of their age and race, body mass Index (BMI), serum levels of 25(OH)D3, blood pressure, total cholesterol, serum Hemoglobin A1C (HBA1C) levels, blood prostate specific antigen (PSA) levels and the number of positive prostate biopsy cores. For these patients, data on comorbidities, including presence of coronary artery disease, hypertension, hypercholesterolemia, diabetes mellitus, depression, and post-traumatic stress disorder and whether they had performed military service were obtained. Finally, data on whether the patients were supplementing with multivitamins or not was gathered.

### 2.2. Differential Prostate Gene Expression between African American and European American Patients

**Figure 1:**
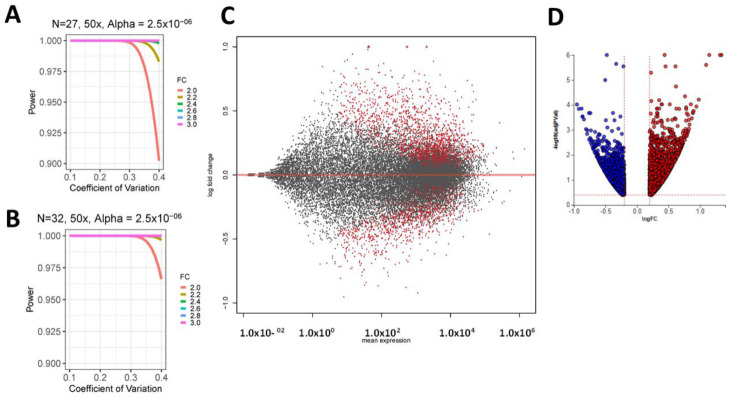
Power analysis plots with power as a function of the co-efficient of variation (CV), using (**A**): 27 and (**B**): 32 replicates respectively to detect FC values ranging from 2.0 to 3.0. Type I error rate is conservatively fixed at α = 2.5 × 10^−6^ (α = 0.05 after Bonferroni Correction). (**C**) MA Plot examining differential gene expression between EA and AA men. The plot visualizes the differences between measurements taken in two samples, by transforming the data onto M (log ratio) and A (mean average) scale. M (*Y*-axis) is plots differential gene expression (log2 (EA/AA). A (*X*-axis) plots transcript abundance (0.5 log2 AA + 0.5 log2 EA). significant transcripts (*q* < 0.1) are highlighted in red. (**D**) Volcano plot depicting the DE genes in the contrast between AA and EA men. The horizontal axis is the log fold change, and the vertical axis is the negative base-10 logarithm of the adj *p*-value or *q*-value. The red-dotted lines represent the threshold. The up-regulated genes (positive log fold change) are shown in red, while the down-regulated genes are blue. AA: African–American; EA: European–American.

We set EA subjects (27 samples) as the control and AA subjects (33 samples) as test cases. These data were analyzed to identify race-associated differences in prostate gene expression. In order to assess if the study had sufficient power, we utilized the sample size tool available in *RNASeqPower* [46]. Power estimation depends on read depth or counts, the associated coefficient of variation (CV) of counts within conditions, fold change (FC) i.e., the effect size, the type I error rate, and the group sample size [47,48]. CVs of 0.1 and 0.4 are typically recommended for inbred (laboratory models) and outbred (human) models, respectively and the sequencing read depth was set at 50 reads per transcript. Figure 1A,B display power as a function of CV, using 27 and 32 replicates respectively to detect FC values ranging from 2.0 to 3.0. Type I error rate was conservatively fixed at α = 2.5 × 10^−6^ (α = 0.05 after Bonferroni Correction). From this analysis we concluded that have at least 90–97% power to detect DE transcripts even for FC = 2 and CV = 0.4 with read counts of at least 50. 

This power analysis is extremely conservative as we identified differentially expressed (DE) transcripts based on false discovery rate (FDR) (Figure 1C,D). Also, our earlier study on a smaller patient cohort was sufficiently powered to uncover differences in prostate gene expression between AA and EA men [28]. FC estimation and significance testing were performed using DESeq2 [49]. As the patient biopsy material was sampled at different times of the year and the sequencing was done using two Illumina flow cells, we included these potential confounders as a batch factor in the DEseq2 analyses. For each gene, DESeq2 provided a FC, and an adjusted *p* (adj-*p*) or *q*-value equal to the smallest FDR incurred. 

**Figure 2:**
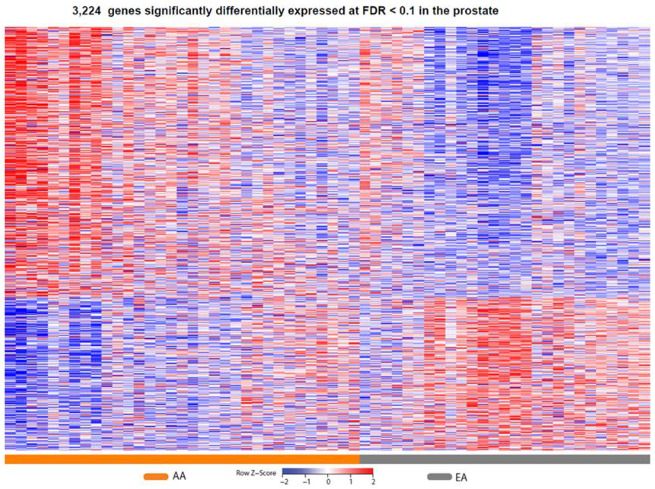
Heat map of gene expression changes by race. Red and blue boxes depict relative over- and under-expression with regard to a reference set as the mid-point between all patients. Only significant transcripts (*q* < 0.1) are shown. AA: African–American; EA: European–American.

When we compared prostate gene expression between AA and EA subjects, this revealed 3277 significantly DE genes with *q* < 0.1 out of a total of 18,797 genes with measured expression (Figure 2).

To identify gene signaling network perturbations in the prostate between EA and AA men we utilized iPathwayGuide [50]. To ensure we included all genes that together enrich gene ontology (GO) terms or biological pathways we relaxed the FDR significance threshold to *q* < 0.4 and required a minimum absolute log fold change in expression of 0.2. Pathways were then scored using the Impact Analysis method. Significant pathways were ranked by their unique pathway-specific adjusted *p*-value. 

Significant (FDR corrected) pathways identified included Nicotine addiction *q* = 7.64 × 10^−6^, Neuroactive ligand-receptor interaction *q* = 1.01 × 10^−3^, Taste transduction *q* = 1.04 × 10^−3^, Olfactory transduction *q* = 1.48 × 10^−3^, ECM-receptor interaction *q* = 8.33 × 10^−3^ and the PI3K-Akt signaling pathway *q* = 1.95 × 10^−3^ (Table 2). 

**Figure 3:**
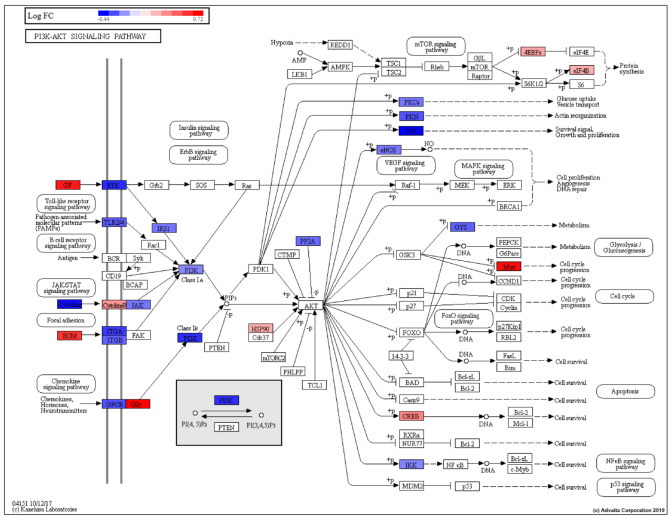
PI3K-Akt signaling pathway (KEGG: 04151) pathway genes showing gene regulation based on EA vs. AA prostate DE analysis (EA is control, AA is test). RED: upregulated, BLUE: downregulated.

The phosphatidylinositol 3′-kinase (PI3K)-Akt signaling pathway is activated by various cellular stimuli and toxins. It regulates basic cellular processes including transcription, translation, proliferation, growth, and survival (Figure 3, Table 2).

Genes belonging to the P13K-Akt signaling pathway that were modestly upregulated among AA compared to EA men included *THBS4*, *CREB3L1*, *TNN*, *COL4A4*, *COL4A3*, *COL2A1*, *FGF12*, *MYC* and *GNG13* (Table S1). Genes belonging to the pathway that were modestly downregulated among AA relative to EA men included *SGK1*, *ANGPT2*, *FGF11*, *IL4*, *IL6*, *ANGPT4*, *THBS2*, *FLT4*, *NTRK2*, *PIK3R6*, *LAMA5*, and *MET*. 

**Figure 4:**
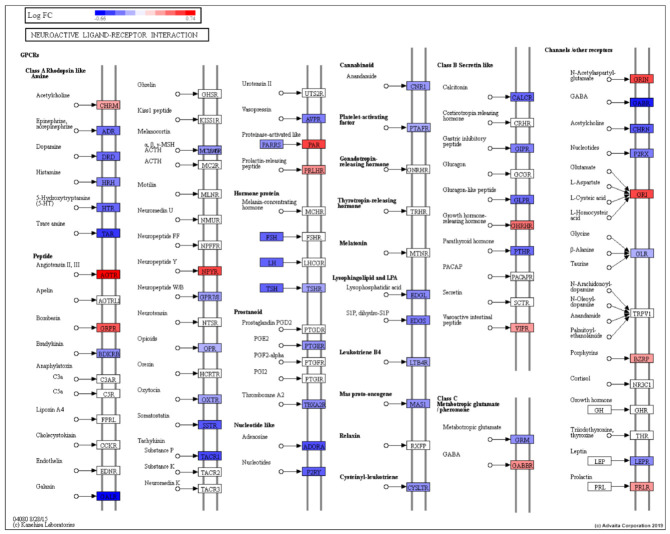
Neuroactive ligand-receptor interaction pathway (KEGG: 04080) showing gene regulation based on EA vs. AA prostate DE analysis (EA is control, AA is test). RED: upregulated, BLUE: downregulated.

The Neuroactive ligand-receptor interaction pathway comprises G-protein coupled receptors, ion channels and ligands that function in modulation of neural plasticity, memory processes, and behavior (Figure 4). 

Genes belonging to the Neuroactive ligand-reception interaction pathway that were modestly upregulated in AA relative to EA men included *AGTR1*, *F2RL2*, *NPY4R* and *GRIN3A*. Genes belonging to this pathway that were modestly downregulated in AA compared to EA men included *GABRP*, *ADORA2B*, *TACR1*, *TAAR1*, *GABRQ* and *GALR1* (Table S2). 

**Figure 5:**
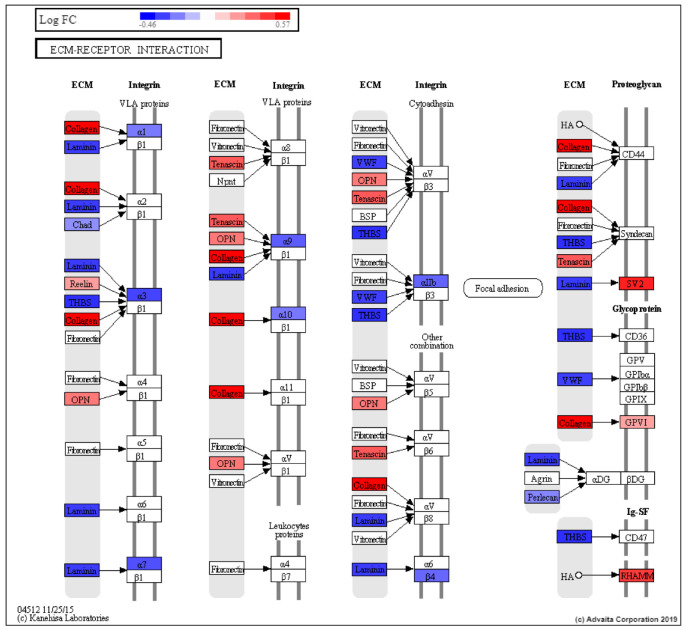
ECM-receptor interaction (KEGG: 04512) DE pathway genes based on EA vs. AA prostate DE analysis (EA is control, AA is test). RED: upregulated, BLUE: downregulated.

The extracellular matrix (ECM) is a complex milieu of functional and structural macromolecules which functions in tissue and organ morphogenesis and maintains the integrity of cell and tissue structure and function (Figure 5). 

Genes belonging to the ECM pathway that were modestly downregulated in EA patients included *LAMA5*, *SV2B*, *THBS2*, *VWF*, *ITGA3*, *ITGA7* and *TNXB* (Table S3). Genes belonging to the ECM pathway that were modestly upregulated in EA relative to AA patients included *THBS4*, *RELN*, *SPP1*, *COL9A2* and *GP6.*

Other pathways included Nicotine addiction, Taste and Olfactory transduction, and Alcoholism. Nicotine is one of the main psychoactive ingredients in tobacco that contributes to the harmful tobacco smoking habit (KEGG: 05033). Taste transduction involves how taste stimuli are detected by taste receptor cells (TRCs). Three distinct cell types are found in mammalians type I, II cells, and type III cells. Type I cells express epithelial sodium channel (ENaC) and mediate the perception of low salt. Type II cells, function in transduction of bitter, sweet and umami taste and are mediated by a PLC-beta/IP3-signaling cascade. Type III cells mediate sour taste via apically located proton-selective ion channels (KEGG: 04742). Olfactory transduction transduces binding of odorant molecules to a receptor into an electrical signal that can be transmitted to the brain (KEGG: 04740). Alcoholism, or dependence on ethanol, is a chronic relapsing disorder with significant unfavorable health outcomes (KEGG: 05034). Cell adhesion molecules (CAMs) are cell surface glycoproteins which play a role in many biological responses the immune response and inflammation (KEGG: 04514). Arginine and proline metabolism are central pathways involved in the biosynthesis of the arginine and proline from glutamate and liked with developmental stage and metastasis (KEGG: 00330). Drug metabolism is mediated by the cytochrome P450 enzymes (KEGG: 00982)

Gene list enrichment analysis was performed using ToppFunn with 3277 DE genes (*q* < 0.1) as input [51]. Of this, 3224 DE genes mapped to known transcripts. This enabled functional enrichment of the gene and uncovered significant differences (Bonferroni corrected *p*-values, *q*) between the two groups (Table S4). 

**Figure 6:**
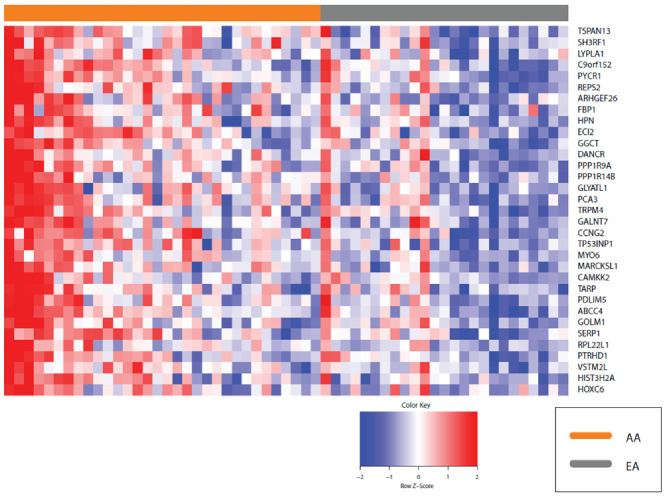
Heat map of gene expression changes in EA men compared with AA men for transcripts that map to the co-expression signature ‘Genes up-regulated in prostate cancer samples’. Red and blue boxes depict relative over- and under-expression with regard to a reference set as the mid-point all patients. Only significant transcripts (*q* < 0.1) are shown.

These included the GO molecular function (MF) terms RNA binding *q* = 4.95 × 10^−10^, and structural constituent of ribosome *q* = 2.79 × 10^−13^, GO biological process (BP) term SRP-dependent co-translational protein targeting to membrane *q* = 4.99 × 10^−16^, the biological pathways translation *q* = 1.42 × 10^−14^ and L13a-mediated translational silencing of Ceruloplasmin expression *q* = 2.34 × 10^−15^; and the co-expression signature ‘Genes up-regulated in prostate cancer samples’ *q* = 1.73 × 10^−21^ (Figure 6, Table S4).

**Figure 7:**
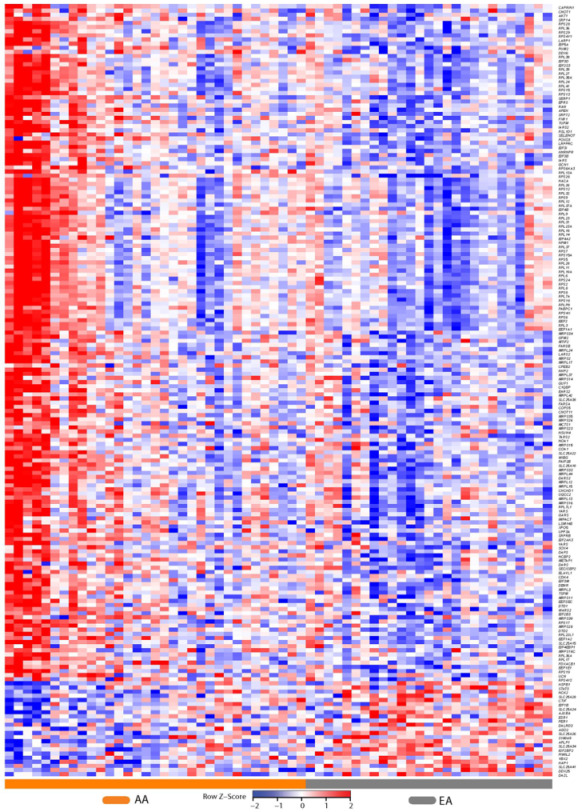
Heatmap of the 187 shared gene signatures derived from the GO terms RNA binding, structural constituent of ribosome, SRP-dependent co-translational protein targeting to membrane and the biological pathways translation, L13a-mediated translational silencing of Ceruloplasmin expression. Red and blue boxes depict relative over- and under-expression with regard to a reference set as the mid-point between all patients. Only significant transcripts (*q* < 0.1) are shown. A cluster of five AA patients is visible at the extreme left of the heatmap.

We next explored the transcripts that mapped to these various GO categories described above and identified 187 transcripts, 50 of which were shared across all these categories and 96 that were unique to translation (Figure 7; Table S5). We plotted the expression of these 187 transcripts in each patient using heat maps. This revealed modest differences between AA and EA patients.

A pattern emerged in which the following transcripts were clearly downregulated in AA relative to EA patients: S100 calcium binding protein (*S100A16*), heat shock protein family B (small) member 1 (*HSPB1*), nuclear RNA export factor 3 (*NXF3*), RANBP2-like and GRIP domain containing 1 (*RGPD1*), apolipoprotein B mRNA editing enzyme catalytic subunit 2 (*APOBEC2*), CUGBP Elav-like family member 3 (*CELF3*), piwi like RNA-mediated gene silencing 2 (*PIWIL2*), RNA binding motif protein 20 (*RBM20*), adenosine deaminase domain containing 2 (*ADAD2*) and ribosomal protein L23a pseudogene 7 (*RPL23AP7*), AHNAK nucleoprotein 2 (*AHNAK2*), solute carrier family 16 member 3 (*SLC16A3*) and Rho guanine nucleotide exchange factor 28 (*ARHGEF28*). 

The heat map revealed a gradation of expression of the 187 transcripts across all patients with extremes observed in both AA and EA patients and a cluster of five AA patients separating from other patients (at the left of the heat-map Figure 7). Many of the prostate transcripts that were upregulated in AA were down regulated in EA and vice versa. 

**Figure 8:**
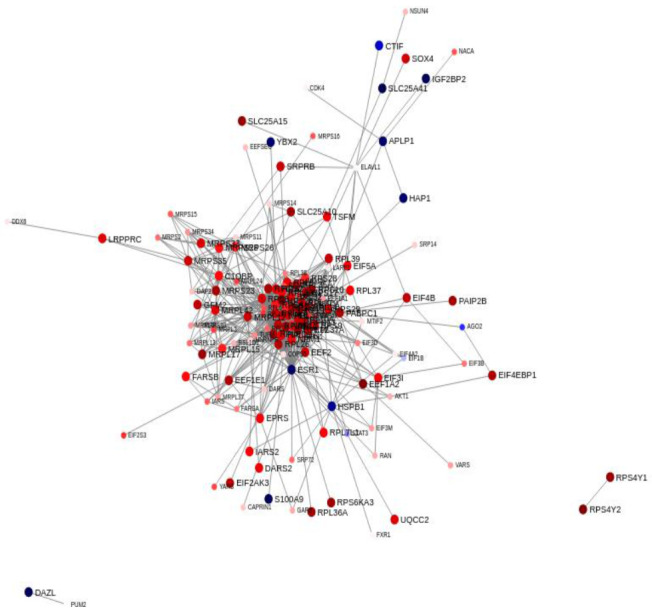
Interaction network of 187 signature genes using The Search Tool for Retrieval of Interacting Genes/Proteins (STRING) Protein-protein interaction (PPI) database. A node represents an individual protein and the edge connecting two nodes represents the relationship between them, i.e., they represent interacting proteins. Nodes with significantly higher degree than other nodes within a system are hub nodes. The nodal color reflects direction and size of regulation in AA men compared to EA men as defined by fold-change values from the experiment. Nodes with no interactions are excluded from the visualization.

To interpret the relationships between the proteins encoded by these 187 transcripts we combined the gene expression data with protein-to-protein interactions (PPIs) interaction data and explored the resulting interactome (Figure 8). This analysis revealed a major PPI network comprised of most of the proteins that constitute this 187 gene signature. The high degree of connectivity observed in amongst these 187 genes is often seen in key signaling molecules which contribute to our understanding the molecular mechanisms of disease. This dense PPI network is suggestive of multiple protein complexes and suggestive of a functional role for these 187 proteins.

We examined the functional roles of these 187 proteins using the ToppGene Suite portal (Table S6) [51]. This revealed that seven of these transcripts *LARP1*, *PUM2*, *PABPC1*, *DDX6*, *DDX25*, *ELAVL1* and *CAPRIN1* mapped to the GO: Cellular Component term cytoplasmic stress granule (*q* = 5.48 × 10^−5^). Forty-four of the encoded proteins were previously identified in yeast two-hybrid screenings of libraries from prostate epithelial cell lines using High Mobility Group B (HMGB) proteins as baits [52]. HMGB proteins are functionally related to cancer progression and *HMGB1* overexpression has been detected in cancerous cells of prostate epithelial origin [53]. Eleven of these transcripts mapped to the GeneSigDB co-expression signature Human Prostate ChoVega05 (*q* = 7.57 × 10^−^^14^) [54]. Next, we examined these 187 proteins using the cBioportal for Cancer Genomics [55,56]. We assessed 4900 samples/4687 patients across 14 studies encompassing prostate adenocarcinoma and prostate cancer and observed that these 187 genes were altered in 1308 (28%) of queried patients and 1383 (28%) of queried samples (Table S7/Figure S1) indicating that this gene signature plays a functional role in prostate cancer progression.

### 2.3. Patient Characteristics and Clinical Phenotype

We wished to compare AA and EA patients across a range of values including age, clinical variables such as blood pressure, body mass index (BMI), serum vitamin D levels, total cholesterol, Hba1c, prostate specific antigen, cancer grade, number of positive biopsy cores, serum DHEA-S and C-reactive protein levels (Table S8). This revealed differences between AA and EA in Vitamin D, Total cholesterol, and Hba1c levels. A comparison of cancer patients based on race revealed significant differences in Vitamin D levels and not surprisingly the number of positive biopsy cores. A comparison of patients without prostate cancer based on race revealed total cholesterol as significantly different between AA and EA (*p* = 0.034) (Table S8).

Subsequently, we wished to determine if clinical phenotypes or patient characteristics correlated with the patient clusters from the heat map of gene expression focusing on the 187 gene signature (Figure 7) and exploring the cluster of five patients that were identified as extremal in the heat map. 

Patient characteristics within the 2 patient clusters in Figure 7 are reported in Table 3. This extreme patient cluster was associated with race, Gleason score, number of positive biopsy punches, triglycerides, and CRP.

The five patients at the extreme cluster in Figure 7 were all AA (100% vs. 52.9% overall cohort, *p* = 0.066). Patients in this extreme gene expression cluster also had higher Gleason scores, a greater number of positive biopsies, higher triglyceride levels, and lower CRP on average.

Patients clustering in this extreme cluster also had lower serum vitamin D levels (serum levels < 20 ng/mL) and reported that they did not supplement with multivitamins, although these associations were not significant. Additionally, these five patients had elevated blood dehydroepiandrosterone sulfate (DHEAS) levels. A summary of the clinical characteristics of these five patients is provided in Table S9. 

#### Sparse Principal Component Analysis

**Figure 9:**
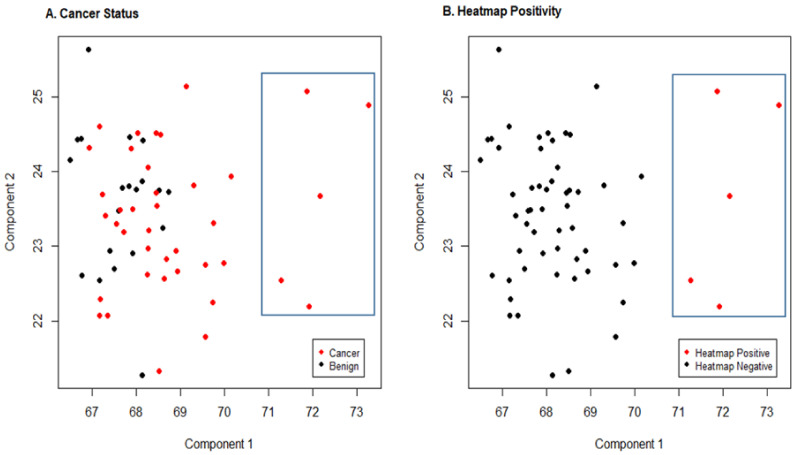
Sparse principal component analysis. Scatter plots of principal component 1 by component 2 from the sparse principal component analysis. (**A**) denotes values of these components by cancer status (red = cancer, black = benign). (**B**) denotes the 5 extreme patients identified by the heat map (Figure 7) in red versus all others in black. The blue rectangle in both plots highlights these five patients.

We next conducted a sparse principal component analysis (PCA) analysis of the expression patterns of the 187 genes using the R *elasticnet* package (Version 1.3) to identify potential subsets of stress signature genes associated with either patient prostate cancer status or with the 5 extreme patients identified by the heatmap (Figure 9). Sparse PCA is a specific technique widely used in statistical analysis and in the analysis of multivariate data sets such as this data. It extends classic PCA for the reduction of dimensionality of data by introducing sparsity structures to the input variables. 

A particular disadvantage of ordinary PCA is that the principal components are usually linear combinations of all input variables. Sparse PCA overcomes this disadvantage by finding linear combinations that contain just a few input variables. Sparse PCA was thus desirable for this analysis as it not only allowed dimensionality reduction but also reduced the number of explicitly used variables. Furthermore, this approach has been previously validated for gene expression data where the number of variables or predictors (*p*) (genes) is typically bigger than the number of samples (*n*) (in this study *n* = 60, *p* = 187) [57]. 

Associations between the sparse PC with cancer status or with the 5 extreme patients were also examined to determine if the 187 gene signature was associated with patient outcome. The first component identified by sparse PCA included 54 of the 187 genes and the second component included 34 of the 187 genes. As noted above GO/pathway analysis of the 187 genes found that these genes map to RNA binding, structural constituent of the ribosome and translation. The majority of the 54 genes included in the first component mapped to translational functionality, though there was significant overlap with RNA binding and structural constituent of the ribosome. 

Cancer status was significantly associated with the first component (*p* = 0.004) in patients who were cancer positive. Of note, 51 of the 54 genes in the first component had positive loadings and only 3 had negative loadings which in general suggests that the majority of genes included in the first component were more highly expressed in patients that were cancer positive. 

We also examined the association of the components with the 5 extreme cancer patients identified by the heat map. The first component was strongly associated with these 5 patients (*p* = 0.000007) with these 5 patients showing the largest value for this component. Figure 9 provides plots of principal component 1 compared to principal component 2 with data highlighted by cancer status (Figure 9A) and for a comparison of the 5 extreme patients vs. all others, defined as heatmap positivity (Figure 9B). The five extreme patients are highlighted on both plots.

## 3. Discussion

An emerging hypothesis about cancer risk and outcomes in the context of racial disparities is that psychological and social stressors impact cellular stress responses [58,59]; however, limited empirical data are available on racial differences in cellular stress responses among men who are at risk for developing prostate cancer. Our prior work assessed transcriptomic differences between EA and AA who had undergone a prostatectomy, following a diagnosis of localized prostate cancer. This revealed AA with higher expression of genes associated with immune response and inflammation [28]. This study exploited RNA-Seq and focused on a cohort of men who had presented either with early-stage prostate cancer or were at risk for prostate cancer. The patient population was comprised of veterans attending the Urology Clinic at the Veterans Administration Medical Center because of an elevated serum level of prostate-specific antigen. At the Charleston VAMC, the breakdown of this population is essentially 50/50 African American/Caucasian. We enrolled 33 AA and 27 EA men, 60 subjects in total, who had undergone a prostate biopsy. Blood samples were acquired from each subject at enrollment. Standard pathology was performed according to protocol. Following the pathology assessment of the prostate biopsy, study subjects were categorized by Gleason score and other clinical parameters. Study tissue samples were placed in sterile tubes, de-identified and flash-frozen until RNA extraction was performed for RNA-Seq library construction. A paired end sequencing approach was undertaken. Differential expression analysis was carried out with DEseq2 and systems level analyses using gene enrichment and pathway impact analyses. Although the sample cohort examined was modest with 60 patients (33 AA and 27 EA), this analysis revealed significant differences between EA and AA, with 3277 genes significantly DE between the two racial groups. 

Analysis of these DE transcripts revealed several significantly impacted pathways between EA and AA based on two forms of evidence over representation analysis, and perturbation analysis [60]. A recent study of gene expression changes in prostate cancer using data present in the Gene Expression Omnibus and The Cancer Genome Atlas databases identified 484 DE genes in prostate cancer compared with adjacent normal tissue. The Neuroactive ligand-receptor interaction signaling pathway was one of the significantly impacted biological pathways between EA and AA [60]. The pathway comprises G-protein coupled receptors, ion channels and ligands which function in modulating neural plasticity, memory processes, and behavior. Previous studies have associated this signaling pathway with the development of bladder cancer and renal cell carcinoma [61,62]. A proteomics approach was undertaken recently by Myers et al. to compare EA and AA men. Similar to our findings they noted that this Neuroactive ligand-receptor interaction signaling pathway was enriched in prostate tumors in AA men [63]. Specific interactions between cells and the ECM are facilitated by integrins and proteoglycans, CD36, and other cell-surface-molecules. A recent study examined gene expression differences in prostate cancer tissue between EA and AA and revealed that the stroma was the site of aggressive changes [64]. 

Castration-resistant prostate cancer is a fatal form of the disease, which develops upon resistance to primary androgen deprivation therapy (ADT). The PI3K-AKT-mTOR signaling axis plays an important role in the development and upkeep of CRPC. This pathway is dysregulated in most advanced prostate cancer and enables cancer cells to overcome androgen deprivation related stress [65,66]. Powell and colleagues examined 639 tumor samples (270 AAM, 369 EAM) and demonstrated linkage of DE genes to roles inflammation and lipid metabolism. The PI3 kinase/Akt pathway is influential in prostate cancer development and is involved in pathway crosstalk interactions. PI3 kinase/Akt is upregulated with insulin resistance and hyperinsulinemia [67].

In prostate cancer increased phosphorylation (activation) of Akt can be used to predict disease recurrence [68,69]. In human prostate cells, Akt is activated by high expression of golgi membrane protein 1 (GOLM1) [70]. Higher expression of GOLM1 exhibited oncogenic effects with the activation of Akt being an oncogenic driver. The use of Akt inhibitors abrogates the oncogenic effects of GOLM1, which suggests a precision medicine target for patients with high GOLM1 expression [70,71,72]. *GOLM1* mRNA expression is up regulated in AA (FC + 1.4, *q* = 0.042) along with myosin VI (*MY06*) (FC + 1.4, *q* = 0.004) compared to EA. Both are golgi related genes that are in the *Genes up regulated in prostate cancer* co-expression signature (Figure 6).

Motor proteins are the engines that perform intracellular transport of proteins along filaments to their target destination [73]. The motor protein MYO6 is associated with transport from the golgi to the cell surface of proteins [74,75]. In prostate cancer tissues, MYO6 and GOLM1 are known to co-locate at the golgi [76]. Additionally, GOLM1 is associated with surface membrane protein transport and recycling growth factor responsive receptor tyrosine kinase (RTK) and epidermal growth factor receptor (EGFR) proteins between the golgi and cell surface in hepatocellular carcinoma [77]. Specifically, it was found that GOLM1 recycles the EGFR and RTK membrane proteins resulting in Akt activation with commensurate oncogenic activity and metastatic progression in hepatocellular carcinoma [77].

Given the higher expression levels of *GOLM1* and *MYO6* transcripts in AA relative to EA men (Figure 6) suggests Akt antagonism as a potential target for AA patients. The differential expression of MYO6 and GOLM1 in AA prostate cancer tissue suggests transport of RTK proteins and the resulting oncogenic activity of Akt might be a good target to treat the AA aggressive prostate cancer phenotype. Future work will involve determining if AA prostate cancer tissues exhibit progression and metastatic potential via activated Akt through RTK recycling mechanisms promoted by MYO6 and GOLM1.

The presence of nicotine addiction, taste and olfactory transduction and alcoholism were among significant pathways reported in Table S4 that are due to pathway crosstalk. This is a phenomenon through which a pathway influences another either directly through common genes or through inter-pathway signals or linkage. The crosstalk phenomenon is well known and has been reported in two landmark papers [78,79]. Olfactory receptors (ORs) are expressed not only in the sensory neurons of the olfactory epithelium, but also in other tissues including prostate where their exact functions remain to be fully elucidated [80].

Co-expression analysis, i.e., investigating the simultaneous expression of two or more genes revealed similarity between the genes DE between EA and AA men in our study to the co-expression signature ‘Genes up regulated in prostate cancer samples’ [81] (Figure 5). Pathway and GO analysis functional enrichment revealed an enrichment of GO terms related to RNA binding, structural constituent of the ribosome, SRP-dependent co-translational protein targeting to membrane, and L13a-mediated translational silencing of Ceruloplasmin expression. Many of the terms relating to RNA binding are suggestive of a primitive stress response, such as those found in lower organisms [82].

Structural constituent of the ribosome is defined as the involvement of molecules that maintain structural integrity of the ribosome, e.g., ribosomal protein or ribosomal RNA. The two ribosomal subunits are comprised of rRNA and protein [83]. Alterations in rRNA/binding proteins lead to alternative polyadenylation which modifies the length of 3′ untranslated regions ultimately shortening them, marking them for degradation and modifying binding sites contained within this region. This phenomenon has become an emerging hallmark of cancer [84,85]. A significant body of evidence indicates that alterations in ribosome biogenesis lead to malignant transformation and progression [86]. Both c-MYC and the components of the PI3K-mTORC1 signaling pathway mediate ribosome biogenesis. The tumor suppressor p53 is activated in response to ribosomal biogenesis stress [86]. 

RNA binding proteins have been shown in numerous studies to have differential expression or mutations leading to various malignancies including prostate cancer [87]. In primitive organisms the contribution of translational regulation to the control of gene expression is significant during stress response. The ability of mammalian cells to modulate global protein synthesis in response to cellular stress is essential for cell survival [88]. While modulation of protein synthesis is mediated by the regulation of eukaryotic initiation and elongation factors, RNA-binding proteins (RBPs) provide a crucial extra layer to post-transcriptional regulation and have a major influence on the robust cellular response to external stress. The stress response in eukaryotic cells often inhibits translation initiation and leads to the formation of cytoplasmic RNA-protein stress granules [89]. 

Ribosomes bind on the endoplasmic reticulum and translate RNA sequences into proteins through biosynthesis. The endoplasmic reticulum and golgi organelles work together to support the translation and transport to the cell surface of membrane proteins. The golgi receives these proteins from the endoplasmic reticulum and sorts them for trafficking to their destination [90]. The Translation, GO:0006412 category contains two genes related to the endoplasmic reticulum that have modestly higher expression in AA compared with EA (Figure 6 and Figure 7): (1) the gene protein kinase-like endoplasmic reticulum kinase (*PERK*) (FC + 1.2, *q* = 0.022), and (2) the stress-associated endoplasmic reticulum protein 1 (*SERP1*) (FC + 1.2, *q* = 0.025). 

Disruption of quality control mechanisms by pathological and physiological mechanisms results in the accumulation of misfolded or unfolded proteins followed by increased ER. PERK is part of the endoplasmic stress sensing system that modulates the unfolded protein response (UPR) [91]. When the endoplasmic reticulum is under stress, PERK inhibits ribosomal translation at the endoplasmic reticulum membrane and target proteins for degradation [92]. *SERP1* is upregulated in response to the UPR and rescues membrane proteins during endoplasmic stress to complete their folding [93,94]. Taken together, the two endoplasmic reticulum stress transcripts are suggestive of a greater stress response in AA compared with EA, with membrane proteins potentially being stabilized during endoplasmic reticulum stress through SERP1. ER stress and related UPR has been recognized as a hallmark for various diseases including cancer, kidney injury, male infertility, and neurodegenerative diseases. Recent animal model and human studies have shown high cholesterol and ER stress as emerging factors involved in disease development [95]. A comparison of benign patients in this study based on race revealed total cholesterol as significantly different between AA and EA (*p* = 0.034). The field of hypercholesterolemia and ER stress and their contribution to disease progression continues to expand.

When we examined the transcripts that were driving functional enrichment of RNA binding, structural constituent of the ribosome, SRP-dependent co-translational protein targeting to membrane, and L13a-mediated translational silencing of Ceruloplasmin expression we noted that many of the genes were shared across these terms (Figure 6). We reduced this to a shared set of 187 gene set that mapped to these functional annotations and represented a differential stress signature between AA and EA. We examined the gene expression patterns in these 187 genes in all the patients in our patient. This highlighted differences in expression between the two racial groups for these 187 genes, on a patient-by-patient basis with many of the transcripts modestly up-regulated (≥1.6) in AA compared to EA and small set modestly down-regulated in AA compared to EA (≤1.6). In addition, we also observed an extremal cluster of five AA patients (8% of the cohort). When we examined the clinical characteristics of these extremal patients, we noted elevated Gleason scores, greater numbers of positive core biopsies, higher triglyceride levels, and lower CRP. We also noted diminished serum Vitamin D levels. This 187 gene expression signature defines these extremal patients with more severe clinical characteristics. We explored protein to protein interactions (PPIs) among these 187 proteins and developed biological networks to reduce the complexity and allow these PPIs to be visualized. This network provided a snapshot of the differences between AA and EA patients and revealed a high degree of interaction among these proteins suggesting a functional role for this differential stress signature (Figure 8). Functional analysis revealed that seven of these transcripts *LARP1*, *PUM2*, *PABPC1*, *DDX6*, *DDX25*, *ELAVL1* and *CAPRIN1* mapped to the GO: Cellular Component term cytoplasmic stress granule. Many of these proteins interact with HMGB1 and HMGB2 whose overexpression is linked to tumor progression, metastasis and poor prognosis [96]. HMGB proteins are localized in the nucleus, cytoplasm and are also secreted to the extracellular milieu following either active secretion by immune cells or passive release from necrotic cells. HMGB proteins are functionally related to cancer progression and *HMGB1* overexpression has been detected in cancerous cells of prostate epithelial origin [53]. Eleven of these transcripts mapped to the GeneSigDB co-expression signature Human Prostate ChoVega05 [54]. This signature derived from pure populations of prostate cancer cells obtained from cancer cells from fresh-frozen prostatectomy tissue. Finally, we examined these 187 proteins using the cBioportal for Cancer Genomics [55,56]. We assessed 4900 samples/4687 patients across 14 studies encompassing prostate adenocarcinoma and prostate cancer and observed that these 187 genes were altered in 1308 (28%) of queried patients and 1383 (28%) of queried samples (Table S7/Figure S1) indicating that this gene signature plays a functional role in prostate cancer progression.

In summary even with the modest size of this patient cohort, this genomic signature derived from a prostate biopsy allowed us to distinguish between AA and EA men. Furthermore, we observed a clear set of five outlier patients within the AA group based on this signature. The clinical characteristics of the five patients were characterized by higher Gleason scores, a greater number of positive biopsies, higher triglyceride levels, and lower CRP.

Limitations of this study were the small patient cohort size, and the fact that RNA- Seq profiling was performed on just one biopsy collected from each patient. Ideally two or more distant core biopsies collected from each patient would be preferable. However, as IRBs are highly protective of study subjects’ safety, a request to obtain two or more distant core biopsies from each study subject for purposes of RNA-Seq would risk denial. Another limitation with this study is that the level of stress could not be assessed a priori because it would be greatly affected by the diagnosis (e.g., the Gleason score), the prognosis, and the family and social implications of the health status of each patient after a complete clinical assessment was performed. Any attempt to anticipate the sub-grouping of study subjects was not feasible and would have resulted in a biased approach to the enrollment process. Another limitation was the lack of sample homogeneity in Gleason scores and tumor grades in our comparisons. Homogeneity of samples is achievable in pre-clinical studies but is a major challenge in human studies such as this one. Final limitations and confounders were sampling of the patient biopsy material at different times and Illumina sequencing with two independent runs and flow cells. We corrected these potential confounders as a batch factor in our data analyses. Future work will focus on continued correlation with longitudinal clinical and transcriptome changes in these patients. This will further elucidate the relative importance of the transcriptional changes we report here.

## 4. Materials and Methods

### 4.1. Human Subjects

This study was authorized by the Institutional Review Board (IRB) at the Medical University of South Carolina (MUSC) and the Research and Development (R&D) Committee of the Ralph H. Johnson VA Medical Center (VAMC), (reference number: Pro00058835). Informed consent was obtained from all subjects involved in the study. Participants in this study were recruited from the Urology Clinic at the Ralph H. Johnson Veteran’s Affairs Medical Center. Men qualified to participate in this study were those who have undergone a medically indicated prostate biopsy procedure because of rising serum levels of the prostate-specific antigen (PSA), or to monitor a low-risk prostate cancer as part of the active surveillance standard of care. This differed from our previous study in which men were enrolled at the time of radical prostatectomy [28]. Patients were enrolled at the time of biopsy and underwent standard allostatic load measurements and completed a questionnaire to assess stress and social determinants. We enrolled 60 subjects (27 EA and 33 AA men), who had undergone a prostate biopsy. Blood samples were acquired from each subject (at enrollment) to measure among other variables serum levels of 25-hydroxyvitamin D3 [25(OH)D3]. A description of the patient demographics and relevant clinical characteristics is in Table 1.

Treatment decisions among the cohort included watchful waiting, PSA Screening, active surveillance, XRT, and surgery. Table 1 provides the characteristics of the subjects, distributions of their age and race, BMI, serum levels of 25(OH)D3, blood pressure, total cholesterol, serum HBA1C levels, blood PSA levels and the number of positive prostate biopsy cores.

For these patients, data on comorbidities, including presence of coronary artery disease, hypertension, hypercholesterolemia, diabetes mellitus, depression and post-traumatic stress disorder were available. Additionally, data on military service duty and era, Vietnam, Post-Vietnam, and Persian Gulf War tours were gathered in addition to determining whether the patients had been exposed to Agent Orange during their tours of duty. Patients were queried as whether they were supplementing with multivitamins or not.

### 4.2. Tissue Sample Procurement and RNA Purification

Standard pathology was performed according to protocol at the Ralph H. Johnson VAMC. Study tissue samples were placed in sterile tubes, de-identified and flash-frozen using liquid nitrogen, and transported to the Genomics Core Facility at MUSC Total RNA was purified using Qiagen RNeasy in accordance with the manufacturer’s instructions. RNA integrity was assessed using the Agilent 2100 Bioanalyzer RNA 6000 Nano Assay chips (Agilent Technologies, Palo Alto, CA, USA).

### 4.3. RNA Sequencing and Analyses

200 ng of total RNA from a biopsy yield of 400–500 ng was used to construct RNA-Seq libraries using the TruSeq RNA Sample Prep Kit (Illumina, San Diego, CA, USA). High throughput sequencing (HTS) was performed using an Illumina HiSeq2500. Each sample was sequenced using a paired end approach to a depth of ~60 million reads or greater. Data were subjected to Illumina quality control (QC) procedures. Secondary analysis was performed as we have previously described using the Genomics Research Platform (OnRamp Bioinformatics, San Diego, CA, USA) [28,97]. The RNA-Seq workflow used to process the data included (1) fastq validation and quality control, (2) read alignment to the human genome (hg19) using the Spliced Transcripts Alignment to a Reference (STAR) Aligner [98], which revealed >93% mapping of the PE reads, (3) extraction of gene-level count data using HTSeq, and (4) differential expression analysis with DEseq2 [47,48,49]. FDR was calculated using the Benjamini-Hochberg multiple testing adjustment procedure [99]. Statistical analysis of pathways and GO terms was performed using this sorted transcript list as we described [100,101] and via gene set enrichment via ToppGene [51]. Venn diagrams were made using BioVenn [102]. In addition, we utilized iPathwayGuide to perform pathway analyses. Pathways were ranked using the Impact Analysis method [50,103,104]. Impact analysis utilizes two kinds of evidence: (i) the over-representation of DE genes from a particular pathway and (ii) the perturbation of that pathway measured by disseminating the measured expression changes across the topology of the pathway [105,106,107,108]. To address power, we used a sample size tool available in RNASeqPower (Figure 1) [46]. R version 4.0.0 software environment was used for all analyses [109]. 

### 4.4. Heat Maps

Heat maps were generated from count data normalized by the variance stabilizing transformation method using DESeq2 version 1.16.1 [49]. For comparison of EA and AA samples unsupervised hierarchical clustering was performed using gplots version 3.0.1. Guided by the hierarchical clustering patient data was subsequently ordered separating the AA from the EA samples and the up-regulated from the down-regulated genes. All heat maps for EA and AA comparisons were made using the same ordering scheme.

### 4.5. Statistical Testing of Patient Characteristics and Clinical Phenotype

Associations between the two patient clusters identified in the heat maps with continuous phenotypes/characteristics were examined using 2 sample *t*-tests or Wilcoxon rank sum tests when applicable and associations with categorical phenotypes were examined using a Fisher’s exact test approach. Given the sample patient size, this analysis was considered exploratory and all associations with a *p* < 0.1 were considered as potentially meaningful. Sparse PCA combines principal component analysis with a penalized regression approach which provides greater consistency in components relative to standard PCA when data are high dimensional [110,111]. Additionally, sparse PCA yields components with non-zero loadings on only a subset of the original predictors, thus simultaneously providing variable selection and dimension reduction [57]. We conducted a sparse PCA analysis of the 187 transcripts we identified as a shared gene signature from Pathway and GO analysis using the R *elasticnet* package (Version 1.3). Associations between the sparse PC with cancer status or with a stress phenotype identified by cluster analysis extreme patients were examined using logistic regression.

### 4.6. Ethics Approval and Consent to Participate

Informed consent was obtained from all subjects involved in the study. This study was authorized by the Institutional Review Board (IRB) at the Medical University of South Carolina (MUSC) and the Research and Development (R&D) Committee of the Ralph H. Johnson VA Medical Center (VAMC). Participants in this study were recruited from the Urology Clinic at the Ralph H. Johnson Veteran’s Affairs Medical Center.

### 4.7. Availability of Data and Materials

The data that support the findings of this study are available at National Center for Biotechnology Information (NCBI) Gene Expression Omnibus (GEO) database; accession number GSE138503; https://www.ncbi.nlm.nih.gov/geo/query/acc.cgi?acc=GSE138503, accessed on 12 October 2021.

## 5. Conclusions

Our goal in this study was to identify the systemic milieu in which prostate cancer develops and potentially progresses, especially as it relates to EA vs. AA. With this objective in mind, we interrogated the prostate transcriptome with a single biopsy core and examined for the first-time gene expression patterns relevant to prostate cancer differences between EA and AA men presenting with early-stage prostate cancer. Comparison across racial groupings uncovered differences in transcriptomic profiles. Transcriptomic analyses indicated a differential stress gene signature between AA and EA which allowed separation of patients with more severe clinical characteristics.

## Figures and Tables

**Table 1:** Patient demographics and measured clinical variables.

Overall *n* = 60				African Americans N (%) 33 (55%)			Caucasians N (%) 27 (45%)		
	Median (IQR)	Minimum	Maximum	Median (IQR)	Minimum	Maximum	Median (IQR)	Minimum	Maximum
**Age, Years**	65 (6)	46	76	65 (5)	46	76	65.5 (7)	46	75
**Blood Pressure, mmHg**	SBP: 135 (33) DBP: 82 (12)	109 62	183 100	SBP: 137 (23) DBP: 84 (16)	109 66	183 100	SBP:130 (34) DBP: 79.5 (12)	112 62	170 98
**Body Mass Index, kg/m^2^**	29.2 (7.6)	17.8	52.1	29.1 (7.5)	14.8	52.1	29.4 (7.4)	22.1	37.4
**Vitamin D (ng/mL)**	28 (11.1)	4.8	68.6	22.6 (18.6)	4.8	63.3	32.5 (14.7)	11.1	68.6
**Total cholesterol (mg/dL)**	189 (51)	85	271	201 (42)	85	271	174.5 (33)	109	257
**HBA1C Level %**	5.6 (0.7)	4.1	10.5	5.7 (0.7)	4.1	10.5	5.45 (0.7)	4.6	9.2
**PSA (μg/L)**	6.21 (3.27)	0.56	31.5	6.12 (2.40)	1.7	31.5	6.47 (3.30)	0.56	20.3
**Grade**	6 (7)	0	9	6 (1)	0	9	0 (6)	0	9
**Number Positive cores**	1 (4)	0	12	3 (5)	0	12	0 (1)	0	4

**Table 2:** Analysis of Impacted Pathways between AA and EA men.

Pathway Name	pv_fdr
Nicotine addiction	7.64 × 10^−6^
Neuroactive ligand-receptor interaction	1.01 × 10^−3^
Taste transduction	1.01 × 10^−3^
Olfactory transduction	1.48 × 10^−3^
Arginine and proline metabolism	1.60 × 10^−3^
ECM-receptor interaction	8.33 × 10^−3^
Cell adhesion molecules (CAMs)	8.80 × 10^−3^
Drug metabolism-cytochrome P450	1.95 × 10^−2^
Alcoholism	1.95 × 10^−2^
PI3K-Akt signaling pathway	1.95 × 10^−2^

**Table 3:** Patient characteristics of the five most extreme AA patients compared to patients (*n* = 54) who did not cluster with these five patients.

Most Extreme AA vs. Others	Others (*n* = 54)	Most Extreme AA (*n* = 5)	*p*
Age, years, mean (SD)	65.4 (6.37)	60.0 (8.32)	0.125
Race, White, *n* (%)	26 (48.1)	0 (0.00)	0.066
BMI, kg/m2, mean (SD)	29.4 (5.93)	30.2 (5.77)	0.775
PSA, (μg/L), median (IQR)	6.16 (3.40)	6.25 (0.70)	0.418
Cancer, Yes, No (%)	34 (63.0)	5 (100.0)	0.156
Gleason Score, median (IQR)	6 (7)	7 (0)	0.046
Number Positive, median (IQR)	1 (3)	6 (5)	0.010
Comorbidities, Yes, *n* (%)			
Coronary Artery Disease	6 (11.1)	0 (0.00)	1.000
Hypertension	35 (64.8)	3 (60.0)	1.000
Hypercholesterolemia	41 (75.9)	3 (60.0)	0.593
Diabetes mellitus	15 (27.8)	2 (40.0)	0.620
Depression	8 (14.8)	1 (20.0)	0.577
Post-Traumatic Stress Disorder	10 (18.5)	2 (40.0)	0.266
Patient Measures, mean (SD)			
SBP	136.0 (17.1)	138.2 (26.6)	0.794
DBP	81.2 (9.06)	85.2 (7.60)	0.349
Heart Rate	74.2 (12.6)	80.2 (16.0)	0.320
LDL	122.8 (36.4)	97.0 (50.1)	0.146
Triglycerides	111.5 (51.5)	141.4 (31.3)	0.073
Total Cholesterol	192.1 (39.7)	176.2 (56.5)	0.413
WHR	0.98 (0.074)	1.00 (0.076)	0.617
A1c	5.73 (0.86)	6.02 (2.57)	0.330
DHEA-S, median (IQR)	114 (91)	279 (173)	0.211
Serum Creatinine	1.04 (0.22)	1.03 (0.28)	0.944
Serum Albumin	3.89 (0.31)	3.86 (0.34)	0.855
C-Reactive Protein	0.50 (0.81)	0.24 (0.11)	0.035
Serum D3 levels	31.0 (14.2)	20.3 (13.3)	0.112
Service Era, *n* (%)			0.757
Vietnam	38 (70.4)	3 (60.0)	
Post-Vietnam	11 (20.4)	1 (20.0)	
Persian Gulf War	5 (9.26)	1 (20.0)	
Agent Orange Exposure, Yes, *n* (%)	10 (18.5)	1 (20.0)	1.000
Multivitamin Supplementation, Yes, *n* (%)	19 (35.2)	0 (0.0)	0.165
Vitamin D Supplementation, Yes, *n* (%)	15 (27.8)	0 (0.0)	0.316
Treatment Decision, *n* (%)			0.291
Watchful Waiting	3 (5.56)	0 (0.00)	
PSA Screening	18 (33.3)	0 (0.00)	
Active Surveillance	19 (35.2)	2 (40.0)	
XRT	10 (18.5)	2 (40.0)	
Surgery	4 (7.41)	1 (20.0)	

## Data Availability

The data that support the findings of this study are available at National Center for Biotechnology Information (NCBI) Gene Expression Omnibus (GEO) database; accession number GSE138503, https://www.ncbi.nlm.nih.gov/geo/query/acc.cgi?acc=GSE138503, accessed on 12 October 2021.

## Supplementary Materials

The following are available online at https://www.mdpi.com/article/10.3390/cancers13205143/s1, Table S1: PI3K-Akt signaling pathway (KEGG: 04151) Pathway Genes, Table S2: Neuroactive ligand-receptor interaction (KEGG: 04080) Pathway Genes, Table S3: ECM-receptor interaction (KEGG: 04512) Pathway Genes, Table S4: Functional enrichment of differences in prostate gene expression between EA and AA subjects, Table S5: 187 Gene Signature differentially expressed between EA and AA subjects, Table S6: Functional enrichment of the 187 Gene Signature, Table S7: cBioPortal analysis of the 187 Gene Signature, Table S8: Univariate differences in patient clinical characteristics, Table S9: Clinical characteristics of the five extremal patients, Figure S1: Analysis of the 187 gene signature using cBioPortal.
