# Supplementary material for: A Systems Approach to Interrogate Gene Expression Patterns in African American Men Presenting with Clinically Localized Prostate Cancer
Source: Cancers (Basel). 2021 Oct 14;13(20):5143. doi: 10.3390/cancers13205143 (PMC8533960; doi:10.3390/cancers13205143)
Supplement: Supplementary file 1 [file cancers-13-05143-s001.zip › Hardiman_et_al_2021_supplemental_MDPI___revision.pdf]

Supplemental Materials

Supplemental Table S1

PI3K-Akt signaling pathway (KEGG: 04151) Pathway Genes.

Supplemental Table S2

Neuroactive ligand-receptor interaction (KEGG: 04080) Pathway Genes.

Supplemental Table S3

ECM-receptor interaction (KEGG: 04512) Pathway Genes.

Supplemental Table S4

Functional enrichment of differences in prostate gene expression between EA and AA subjects.

Supplemental Table S5

187 Gene Signature differentially expressed between EA and AA subjects.

Supplemental Table S6

Functional enrichment of the 187 Gene Signature.

Supplemental Table S7

cBioPortal analysis of the 187 Gene Signature.

Supplemental Table S8

Univariate differences in patient clinical characteristics.

Supplemental Table S9

Clinical characteristics of the five extremal patients.

Supplemental Figure 1

Analysis of the 187 gene signature using cBioPortal

Supplemental Table S1

PI3K-Akt signaling pathway (KEGG: 04151)				
**symbol**	**entrez**	**logfc**	**adjpv***	**linear FC**
IKBKG	8517	-0.21437	2.86E-06	-1.16
LAMA5	3911	-0.35661	0.000408	-1.28
EIF4B	1975	0.222302	0.001843	1.17
MYC	4609	0.684241	0.002596	1.61
GNG13	51764	0.716084	0.004221	1.64
ITGA3	3675	-0.34056	0.005339	-1.27
FLT4	2324	-0.37325	0.006005	-1.30
FGF12	2257	0.64987	0.010108	1.57
PKN3	29941	-0.29983	0.011717	-1.23
PPP2R2A	5520	0.211479	0.01252	1.16
IL4R	3566	-0.2727	0.013869	-1.21
FGF11	2256	-0.43209	0.01788	-1.35
COL2A1	1280	0.567253	0.019076	1.48
COL4A4	1286	0.381646	0.023704	1.30
COL4A3	1285	0.396176	0.024512	1.32
LAMB2	3913	-0.21017	0.033702	-1.16
LPAR2	9170	0.201344	0.034622	1.15
ANGPT2	285	-0.4429	0.037598	-1.36
FGFR2	2263	-0.29298	0.038476	-1.23
VWF	7450	-0.34847	0.040538	-1.27
IRS1	3667	-0.23985	0.052486	-1.18
CREB3L1	90993	0.348802	0.052863	1.27
SGK1	6446	-0.44368	0.053718	-1.36
ITGA10	8515	-0.24684	0.055965	-1.19
MET	4233	-0.35173	0.065716	-1.28
CSF1	1435	-0.27628	0.072675	-1.21
PDGFRA	5156	-0.26093	0.076869	-1.20
ITGB4	3691	-0.27569	0.077562	-1.21
ITGA7	3679	-0.31864	0.078729	-1.25
KDR	3791	-0.24711	0.079031	-1.19
COL6A1	1291	-0.2783	0.082111	-1.21
COL6A2	1292	-0.26849	0.083799	-1.20
EIF4EBP1	1978	0.251608	0.083829	1.19

THBS2	7058	-0.38078	0.085981	-1.30
HSP90B1	7184	0.223312	0.088755	1.17
TNXB	7148	-0.30845	0.095009	-1.24
PIK3R6	146850	-0.36466	0.095552	-1.29
GNB3	2784	-0.28503	0.095995	-1.22
PRKCA	5578	-0.23967	0.10018	-1.18
IL4	3565	-0.41787	0.107244	-1.34
ANGPT4	51378	-0.39249	0.111252	-1.31
IL6	3569	-0.40325	0.116927	-1.32
NTRK2	4915	-0.36981	0.117461	-1.29
ITGA9	3680	-0.29648	0.120306	-1.23
NTRK1	4914	-0.34117	0.123507	-1.27
NOS3	4846	-0.23475	0.125135	-1.18
PRLR	5618	0.298222	0.127059	1.23
HGF	3082	-0.32834	0.129311	-1.26
EPOR	2057	0.221308	0.132139	1.17
LAMB3	3914	-0.29729	0.135987	-1.23
ITGA2B	3674	-0.28357	0.140323	-1.22
FLT1	2321	-0.20711	0.141834	-1.15
LAMA3	3909	-0.23411	0.146017	-1.18
COL1A1	1277	-0.23868	0.152028	-1.18
TNN	63923	0.369779	0.157007	1.29
ITGA1	3672	-0.24141	0.162141	-1.18
IGF1	3479	0.272918	0.17219	1.21
CHAD	1101	-0.20327	0.177767	-1.15
EPHA2	1969	-0.26595	0.184177	-1.20
LAMA4	3910	-0.20112	0.187402	-1.15
NTF4	4909	-0.22145	0.190429	-1.17
VEGFA	7422	-0.28381	0.200563	-1.22
FGFR3	2261	-0.23292	0.204355	-1.18
LAMC3	10319	-0.25849	0.207883	-1.20
LPAR4	2846	-0.30214	0.212483	-1.23
COL4A6	1288	-0.23857	0.21301	-1.18
TLR2	7097	-0.28197	0.217326	-1.22
THBS4	7060	0.312586	0.228792	1.24
KIT	3815	-0.20629	0.243568	-1.15
PPP2R2B	5521	-0.25892	0.254433	-1.20
RELN	5649	0.216024	0.260706	1.16
SPP1	6696	0.297591	0.264992	1.23
PIK3CD	5293	-0.20716	0.271843	-1.15
FGF10	2255	-0.25834	0.271865	-1.20

GNG3	2785	0.219653	0.285354	1.16
COL9A2	1298	0.238746	0.285687	1.18
EFNA5	1946	-0.21035	0.290755	-1.16
GYS2	2998	-0.27625	0.292976	-1.21
NTF3	4908	-0.20765	0.29765	-1.15
JAK3	3718	-0.24024	0.330159	-1.18
FGF20	26281	-0.25641	0.335783	-1.19
GNG8	94235	-0.23643	0.378291	-1.18
CSF3	1440	-0.22177	0.388395	-1.17
FLT3	2322	-0.2311	0.392357	-1.17
IL7R	3575	-0.22889	0.397705	-1.17

*The raw p-values obtained from the DEseq2 analyses using the Wald test were corrected for multiple testing using the Benjamini and Hochberg method and presented as an adjpv.

Supplemental Table S2

Neuroactive ligand-receptor interaction (KEGG: 04080)				
symbol	entrez	logfc	adjpv*	linear FC
AGTR1	185	0.737259	0.001827	1.67
F2RL2	2151	0.552181	0.026245	1.47
NPY4R	5540	0.54124	0.029276	1.46
GRIN3A	116443	0.536148	0.019504	1.45
GRPR	2925	0.486587	0.055335	1.40
F2RL1	2150	0.481602	0.025608	1.40
GABRA3	2556	0.416834	0.109854	1.33
NPY2R	4887	0.407444	0.10093	1.33
GHRHR	2692	0.399768	0.124978	1.32
SSTR1	6751	0.376604	0.132771	1.30
GABRA1	2554	0.353279	0.131396	1.28
GPR156	165829	0.320198	0.129783	1.25
PRLHR	2834	0.319184	0.185696	1.25
PRLR	5618	0.298222	0.127059	1.23
VIPR1	7433	0.288326	0.038936	1.22
TSPO	706	0.284525	0.001995	1.22
GRIA3	2892	0.284175	0.217231	1.22
CHRM3	1131	0.257315	0.328561	1.20
CHRNA4	1137	0.254186	0.339518	1.19
GABRA5	2558	0.253902	0.342101	1.19
GZMA	3001	0.238317	0.301778	1.18
GABRB3	2562	0.224255	0.362616	1.17
LPAR2	9170	0.201344	0.034622	1.15
OPRL1	4987	-0.20216	0.225355	-1.15
GRIN2D	2906	-0.20535	0.367593	-1.15
GRIN3B	116444	-0.20568	0.258537	-1.15
GLRB	2743	-0.20781	0.140323	-1.15
PTGER2	5732	-0.2182	0.2775	-1.16
PTAFR	5724	-0.22036	0.326468	-1.17
ADRA1D	146	-0.22139	0.367549	-1.17
LTB4R2	56413	-0.22543	0.077618	-1.17
GRIK1	2897	-0.22763	0.345416	-1.17
GABRA2	2555	-0.22833	0.393108	-1.17

CHRM4	1132	-0.22938	0.383619	-1.17
TSHR	7253	-0.23306	0.310901	-1.18
GRIN2A	2903	-0.23489	0.374608	-1.18
GRIA2	2891	-0.23593	0.377616	-1.18
OXTR	5021	-0.23928	0.37369	-1.18
HTR2C	3358	-0.2397	0.37982	-1.18
CHRNB4	1143	-0.24821	0.359352	-1.19
GABBR2	9568	-0.25017	0.356613	-1.19
GRM8	2918	-0.2508	0.334154	-1.19
NPBWR1	2831	-0.25101	0.356605	-1.19
GRIA1	2890	-0.26165	0.333798	-1.20
GRM2	2912	-0.2627	0.116927	-1.20
CNR1	1268	-0.26623	0.270793	-1.20
P2RX6	9127	-0.26734	0.275586	-1.20
S1PR1	1901	-0.26862	0.048492	-1.20
MC1R	4157	-0.27278	0.02163	-1.21
BDKRB1	623	-0.27411	0.300691	-1.21
PTGER3	5733	-0.2747	0.250234	-1.21
MAS1	4142	-0.27918	0.295815	-1.21
CYSLTR1	10800	-0.28027	0.153197	-1.21
GRIK2	2898	-0.28198	0.167237	-1.22
CTSG	1511	-0.28257	0.274717	-1.22
GPR83	10888	-0.28565	0.235556	-1.22
GRM3	2913	-0.28758	0.270938	-1.22
CHRNB2	1141	-0.29109	0.194316	-1.22
BDKRB2	624	-0.2919	0.190115	-1.22
DRD1	1812	-0.29297	0.139983	-1.23
TBXA2R	6915	-0.29872	0.106044	-1.23
HTR7	3363	-0.29902	0.20151	-1.23
LPAR4	2846	-0.30214	0.212483	-1.23
LEPR	3953	-0.30237	0.04699	-1.23
CHRNA7	1139	-0.31574	0.067444	-1.24
AVPR2	554	-0.32049	0.047935	-1.25
HRH1	3269	-0.32335	0.126788	-1.25
GIPR	2696	-0.33075	0.098622	-1.26
AVPR1A	552	-0.33307	0.169455	-1.26
P2RX2	22953	-0.33768	0.10349	-1.26
S1PR5	53637	-0.33801	0.117393	-1.26
HTR1B	3351	-0.33948	0.182629	-1.27
ADORA3	140	-0.36537	0.074751	-1.29
ADRA2B	151	-0.37088	0.085038	-1.29

GLP2R	9340	-0.38062	0.141443	-1.30
PTH1R	5745	-0.38248	0.055175	-1.30
GABRE	2564	-0.3855	0.065253	-1.31
DRD2	1813	-0.38632	0.123719	-1.31
GABRR1	2569	-0.39301	0.131855	-1.31
HTR1F	3355	-0.39516	0.115922	-1.32
CALCRL	10203	-0.39876	0.010339	-1.32
CGA	1081	-0.40681	0.102152	-1.33
P2RY2	5029	-0.4169	0.065862	-1.34
CHRNA3	1136	-0.42939	0.070275	-1.35
SSTR5	6755	-0.43797	0.05196	-1.35
GABRP	2568	-0.4478	0.08339	-1.36
ADORA2B	136	-0.46144	0.03945	-1.38
TACR1	6869	-0.47698	0.029222	-1.39
TAAR1	134864	-0.52462	0.03558	-1.44
GABRQ	55879	-0.5921	0.017826	-1.51
GALR1	2587	-0.66066	0.004028	-1.58

*The raw p-values obtained from the DEseq2 analyses using the Wald test were corrected for multiple testing using the Benjamini and Hochberg method and presented as an adjpv.

Supplemental Table S3

ECM-receptor interaction (KEGG: 04512) symbol	entrez	logfc	adjpv*	linear FC
LAMA5	3911	-0.35661	0.000408	-1.28
ITGA3	3675	-0.34056	0.005339	-1.27
SV2C	22987	0.49535	0.01788	1.41
COL2A1	1280	0.567253	0.019076	1.48
COL4A4	1286	0.381646	0.023704	1.30
COL4A3	1285	0.396176	0.024512	1.32
LAMB2	3913	-0.21017	0.033702	-1.16
VWF	7450	-0.34847	0.040538	-1.27
ITGA10	8515	-0.24684	0.055965	-1.19
HMMR	3161	0.436733	0.065557	1.35
SV2B	9899	-0.46348	0.072763	-1.38
ITGB4	3691	-0.27569	0.077562	-1.21
ITGA7	3679	-0.31864	0.078729	-1.25
COL6A1	1291	-0.2783	0.082111	-1.21
COL6A2	1292	-0.26849	0.083799	-1.20
THBS2	7058	-0.38078	0.085981	-1.30
TNXB	7148	-0.30845	0.095009	-1.24
ITGA9	3680	-0.29648	0.120306	-1.23
LAMB3	3914	-0.29729	0.135987	-1.23
ITGA2B	3674	-0.28357	0.140323	-1.22
LAMA3	3909	-0.23411	0.146017	-1.18
COL1A1	1277	-0.23868	0.152028	-1.18
TNN	63923	0.369779	0.157007	1.29
ITGA1	3672	-0.24141	0.162141	-1.18
HSPG2	3339	-0.22205	0.164337	-1.17
CHAD	1101	-0.20327	0.177767	-1.15
LAMA4	3910	-0.20112	0.187402	-1.15
LAMC3	10319	-0.25849	0.207883	-1.20
COL4A6	1288	-0.23857	0.21301	-1.18
THBS4	7060	0.312586	0.228792	1.24
RELN	5649	0.216024	0.260706	1.16
SPP1	6696	0.297591	0.264992	1.23
COL9A2	1298	0.238746	0.285687	1.18
GP6	51206	0.205907	0.39587	1.15

*The raw p-values obtained from the DEseq2 analyses using the Wald test were corrected for multiple testing using the Benjamini and Hochberg method and presented as an adjpv.

Supplemental Table S4. Functional enrichment of differences in prostate gene expression between EA and AA subjects.

Gene Ontology ID<br>Molecular Function	Name	Bonferroni<br>q-value
GO:0003735	structural constituent of ribosome	2.79E-13
GO:0003723	RNA binding	4.95E-10
GO:0019843	rRNA binding	1.96E-04
GO:0005198	structural molecule activity	2.05E-02
GO:0008536	Ran GTPase binding	1.10E-01
**Biological Process**	**Name**	**q-value**
GO:1901566	organonitrogen compound biosynthetic process	1.14E-16
GO:0006614	SRP-dependent cotranslational protein targeting to membrane	4.99E-16
GO:0006613	cotranslational protein targeting to membrane	5.33E-15
GO:0043043	peptide biosynthetic process	5.61E-15
GO:0006412	translation	1.42E-14
GO:0045047	protein targeting to ER	5.99E-14
GO:0043604	amide biosynthetic process	1.15E-13
GO:0000184	nuclear-transcribed mRNA catabolic process, nonsense-mediated decay	1.78E-13
GO:0072599	establishment of protein localization to endoplasmic reticulum	5.10E-13
GO:0006413	translational initiation	6.47E-12
GO:0070972	protein localization to endoplasmic reticulum	2.68E-11
GO:0006518	peptide metabolic process	3.83E-11
GO:0019083	viral transcription	4.56E-11
GO:0043603	cellular amide metabolic process	7.91E-11
**Pathway ID<br>REACTOME**	**Name**	**q-value**
1268691	Peptide chain elongation	5.22E-17
1339149	Selenoamino acid metabolism	9.42E-17
1268690	Eukaryotic Translation Elongation	1.75E-16
1339156	Selenocysteine synthesis	3.58E-16
1269120	Viral mRNA Translation	3.90E-16
1268688	L13a-mediated translational silencing of Ceruloplasmin expression	2.34E-15
1268680	Cap-dependent Translation Initiation	4.07E-15
1268679	Eukaryotic Translation Initiation	4.07E-15
1268692	Eukaryotic Translation Termination	4.53E-15
1268681	Formation of a pool of free 40S subunits	5.04E-15
1268686	GTP hydrolysis and joining of the 60S ribosomal subunit	1.32E-14
1269716	Nonsense-Mediated Decay (NMD)	1.86E-13
1269109	Influenza Life Cycle	2.39E-13
1269115	Influenza Viral RNA Transcription and Replication	5.73E-13
1268678	Translation	7.09E-13
1268689	SRP-dependent cotranslational protein targeting to membrane	2.52E-12

1269108	Influenza Infection	3.85E-12
1270158	Metabolism of amino acids and derivatives	1.91E-11
1383086	Major pathway of rRNA processing in the nucleolus and cytosol	5.57E-10
1383085	rRNA processing	7.42E-10
1427846	rRNA processing in the nucleus and cytosol	1.60E-09
1269649	Gene Expression	3.12E-09
**Coexpression ID**	**Name**	**q-value**
M4619	Genes up-regulated in prostate cancer samples.	1.73E-21
M19148	Genes up-regulated in prostate cancer vs benign prostate tissue	4.54E-09

Supplemental Table S5 - 187 Gene Signature DE between EA and AA men.

Gene Symbol	Gene description	Gene ID
AGO2	AGO2 (argonaute RISC catalytic component 2)	27161
AJUBA	AJUBA (ajuba LIM protein)	84962
AKT1	AKT1 (AKT serine/threonine kinase 1)	207
APEH	APEH (acylaminoacyl-peptide hydrolase)	327
APLP1	APLP1 (amyloid beta precursor like protein 1)	333
C1QBP	C1QBP (complement C1q binding protein)	708
CAPRIN1	CAPRIN1 (cell cycle associated protein 1)	4076
CDK4	CDK4 (cyclin dependent kinase 4)	1019
CHCHD1	CHCHD1 (coiled-coil-helix-coiled-coil-helix domain containing 1)	118487
CNOT1	CNOT1 (CCR4-NOT transcription complex subunit 1)	23019
CNOT11	CNOT11 (CCR4-NOT transcription complex subunit 11)	55571
COA1	COA1 (cytochrome c oxidase assembly factor 1 homolog)	55744
COPS5	COPS5 (COP9 signalosome subunit 5)	10987
CPEB2	CPEB2 (cytoplasmic polyadenylation element binding protein 2)	132864
CTIF	CTIF (cap binding complex dependent translation initiation factor)	9811
DALRD3	DALRD3 (DALR anticodon binding domain containing 3)	55152
DAP3	DAP3 (death associated protein 3)	7818
DARS	DARS (aspartyl-tRNA synthetase)	1615
DARS2	DARS2 (aspartyl-tRNA synthetase 2, mitochondrial)	55157
DAZL	DAZL (deleted in azoospermia like)	1618
DDX25	DDX25 (DEAD-box helicase 25)	29118
DDX6	DDX6 (DEAD-box helicase 6)	1656
DENR	DENR (density regulated re-initiation and release factor)	8562
DTD1	DTD1 (D-tyrosyl-tRNA deacylase 1)	92675
DTD2	DTD2 (D-tyrosyl-tRNA deacylase 2 (putative))	112487
EARS2	EARS2 (glutamyl-tRNA synthetase 2, mitochondrial)	124454
EEF1A1	EEF1A1 (eukaryotic translation elongation factor 1 alpha 1)	1915
EEF1A2	EEF1A2 (eukaryotic translation elongation factor 1 alpha 2)	1917
EEF1E1	EEF1E1 (eukaryotic translation elongation factor 1 epsilon 1)	9521
EEF2	EEF2 (eukaryotic translation elongation factor 2)	1938
EEFSEC	EEFSEC (eukaryotic elongation factor, selenocysteine-tRNA specific)	60678
EIF1B	EIF1B (eukaryotic translation initiation factor 1B)	10289
EIF2AK3	EIF2AK3 (eukaryotic translation initiation factor 2 alpha kinase 3)	9451
EIF2B3	EIF2B3 (eukaryotic translation initiation factor 2B subunit gamma)	8891
EIF2S3	EIF2S3 (eukaryotic translation initiation factor 2 subunit gamma)	1968
EIF3B	EIF3B (eukaryotic translation initiation factor 3 subunit B)	8662
EIF3D	EIF3D (eukaryotic translation initiation factor 3 subunit D)	8664
EIF3I	EIF3I (eukaryotic translation initiation factor 3 subunit I)	8668
EIF3M	EIF3M (eukaryotic translation initiation factor 3 subunit M)	10480
EIF4A2	EIF4A2 (eukaryotic translation initiation factor 4A2)	1974
EIF4B	EIF4B (eukaryotic translation initiation factor 4B)	1975
EIF4EBP1	EIF4EBP1 (eukaryotic translation initiation factor 4E binding protein 1)	1978
EIF5A	EIF5A (eukaryotic translation initiation factor 5A)	1984
ELAVL1	ELAVL1 (ELAV like RNA binding protein 1)	1994
EPRS	EPRS (glutamyl-prolyl-tRNA synthetase)	2058
ESR1	ESR1 (estrogen receptor 1)	2099
FARSA	FARSA (phenylalanyl-tRNA synthetase subunit alpha)	2193
FARSB	FARSB (phenylalanyl-tRNA synthetase subunit beta)	10056
FDXACB1	FDXACB1 (ferredoxin-fold anticodon binding domain containing 1)	91893

FOXO3	FOXO3 (forkhead box O3)	2309
FXR1	FXR1 (FMR1 autosomal homolog 1)	8087
GARS	GARS (glycyl-tRNA synthetase)	2617
GCN1	GCN1 (GCN1, eIF2 alpha kinase activator homolog)	10985
GFM2	GFM2 (G elongation factor mitochondrial 2)	84340
GUF1	GUF1 (GUF1 homolog, GTPase)	60558
HAP1	HAP1 (huntingtin associated protein 1)	9001
HNRNPR	HNRNPR (heterogeneous nuclear ribonucleoprotein R)	10236
HSPB1	HSPB1 (heat shock protein family B (small) member 1)	3315
IARS	IARS (isoleucyl-tRNA synthetase)	3376
IARS2	IARS2 (isoleucyl-tRNA synthetase 2, mitochondrial)	55699
IGF2BP2	IGF2BP2 (insulin like growth factor 2 mRNA binding protein 2)	10644
IMPACT	IMPACT (impact RWD domain protein)	55364
LARP1	LARP1 (La ribonucleoprotein domain family member 1)	23367
LARS2	LARS2 (leucyl-tRNA synthetase 2, mitochondrial)	23395
LRPPRC	LRPPRC (leucine rich pentatricopeptide repeat containing)	10128
LSM14B	LSM14B (LSM family member 14B)	149986
MCTS1	MCTS1 (MCTS1 re-initiation and release factor)	28985
METAP1	METAP1 (methionyl aminopeptidase 1)	23173
MRPL12	MRPL12 (mitochondrial ribosomal protein L12)	6182
MRPL13	MRPL13 (mitochondrial ribosomal protein L13)	28998
MRPL15	MRPL15 (mitochondrial ribosomal protein L15)	29088
MRPL17	MRPL17 (mitochondrial ribosomal protein L17)	63875
MRPL24	MRPL24 (mitochondrial ribosomal protein L24)	79590
MRPL3	MRPL3 (mitochondrial ribosomal protein L3)	11222
MRPL37	MRPL37 (mitochondrial ribosomal protein L37)	51253
MRPL42	MRPL42 (mitochondrial ribosomal protein L42)	28977
MRPL44	MRPL44 (mitochondrial ribosomal protein L44)	65080
MRPS11	MRPS11 (mitochondrial ribosomal protein S11)	64963
MRPS14	MRPS14 (mitochondrial ribosomal protein S14)	63931
MRPS15	MRPS15 (mitochondrial ribosomal protein S15)	64960
MRPS16	MRPS16 (mitochondrial ribosomal protein S16)	51021
MRPS18C	MRPS18C (mitochondrial ribosomal protein S18C)	51023
MRPS2	MRPS2 (mitochondrial ribosomal protein S2)	51116
MRPS23	MRPS23 (mitochondrial ribosomal protein S23)	51649
MRPS26	MRPS26 (mitochondrial ribosomal protein S26)	64949
MRPS28	MRPS28 (mitochondrial ribosomal protein S28)	28957
MRPS30	MRPS30 (mitochondrial ribosomal protein S30)	10884
MRPS33	MRPS33 (mitochondrial ribosomal protein S33)	51650
MRPS34	MRPS34 (mitochondrial ribosomal protein S34)	65993
MRPS35	MRPS35 (mitochondrial ribosomal protein S35)	60488
MTIF2	MTIF2 (mitochondrial translational initiation factor 2)	4528
NACA	NACA (nascent polypeptide associated complex subunit alpha)	4666
NCBP2	NCBP2 (nuclear cap binding protein subunit 2)	22916
NCK2	NCK2 (NCK adaptor protein 2)	8440
NHP2	NHP2 (NHP2 ribonucleoprotein)	55651
NOA1	NOA1 (nitric oxide associated 1)	84273
NPM1	NPM1 (nucleophosmin 1)	4869
NSUN4	NSUN4 (NOP2/Sun RNA methyltransferase family member 4)	387338
PABPC1	PABPC1 (poly(A) binding protein cytoplasmic 1)	26986
PAIP2B	PAIP2B (poly(A) binding protein interacting protein 2B)	400961
PER1	PER1 (period circadian regulator 1)	5187

PIWIL2	PIWIL2 (piwi like RNA-mediated gene silencing 2)	55124
PUM2	PUM2 (pumilio RNA binding family member 2)	23369
RAN	RAN (RAN, member RAS oncogene family)	5901
RPL10A	RPL10A (ribosomal protein L10a)	4736
RPL11	RPL11 (ribosomal protein L11)	6135
RPL12	RPL12 (ribosomal protein L12)	6136
RPL13A	RPL13A (ribosomal protein L13a)	23521
RPL14	RPL14 (ribosomal protein L14)	9045
RPL17	RPL17 (ribosomal protein L17)	6139
RPL18	RPL18 (ribosomal protein L18)	6141
RPL22L1	RPL22L1 (ribosomal protein L22 like 1)	200916
RPL23	RPL23 (ribosomal protein L23)	9349
RPL23A	RPL23A (ribosomal protein L23a)	6147
RPL24	RPL24 (ribosomal protein L24)	6152
RPL26	RPL26 (ribosomal protein L26)	6154
RPL27	RPL27 (ribosomal protein L27)	6155
RPL28	RPL28 (ribosomal protein L28)	6158
RPL3	RPL3 (ribosomal protein L3)	6122
RPL31	RPL31 (ribosomal protein L31)	6160
RPL32	RPL32 (ribosomal protein L32)	6161
RPL35A	RPL35A (ribosomal protein L35a)	6165
RPL36	RPL36 (ribosomal protein L36)	25873
RPL36A	RPL36A (ribosomal protein L36a)	6173
RPL37	RPL37 (ribosomal protein L37)	6167
RPL37A	RPL37A (ribosomal protein L37a)	6168
RPL38	RPL38 (ribosomal protein L38)	6169
RPL39	RPL39 (ribosomal protein L39)	6170
RPL41	RPL41 (ribosomal protein L41)	6171
RPL6	RPL6 (ribosomal protein L6)	6128
RPL7A	RPL7A (ribosomal protein L7a)	6130
RPL7L1	RPL7L1 (ribosomal protein L7 like 1)	285855
RPL8	RPL8 (ribosomal protein L8)	6132
RPL9	RPL9 (ribosomal protein L9)	6133
RPLP0	RPLP0 (ribosomal protein lateral stalk subunit P0)	6175
RPS10	RPS10 (ribosomal protein S10)	6204
RPS12	RPS12 (ribosomal protein S12)	6206
RPS13	RPS13 (ribosomal protein S13)	6207
RPS15	RPS15 (ribosomal protein S15)	6209
RPS15A	RPS15A (ribosomal protein S15a)	6210
RPS17	RPS17 (ribosomal protein S17)	6218
RPS18	RPS18 (ribosomal protein S18)	6222
RPS2	RPS2 (ribosomal protein S2)	6187
RPS24	RPS24 (ribosomal protein S24)	6229
RPS26	RPS26 (ribosomal protein S26)	6231
RPS28	RPS28 (ribosomal protein S28)	6234
RPS29	RPS29 (ribosomal protein S29)	6235
RPS4X	RPS4X (ribosomal protein S4 X-linked)	6191
RPS4Y1	RPS4Y1 (ribosomal protein S4 Y-linked 1)	6192
RPS4Y2	RPS4Y2 (ribosomal protein S4 Y-linked 2)	140032
RPS5	RPS5 (ribosomal protein S5)	6193
RPS6	RPS6 (ribosomal protein S6)	6194
RPS6KA3	RPS6KA3 (ribosomal protein S6 kinase A3)	6197

RPS7	RPS7 (ribosomal protein S7)	6201
RPS8	RPS8 (ribosomal protein S8)	6202
RPS9	RPS9 (ribosomal protein S9)	6203
RSL1D1	RSL1D1 (ribosomal L1 domain containing 1)	26156
S100A9	S100A9 (S100 calcium binding protein A9)	6280
SECISBP2	SECISBP2 (SECIS binding protein 2)	79048
SELENOT	SELENOT (selenoprotein T)	51714
SERP1	SERP1 (stress associated endoplasmic reticulum protein 1)	27230
SLC25A10	SLC25A10 (solute carrier family 25 member 10)	1468
SLC25A15	SLC25A15 (solute carrier family 25 member 15)	10166
SLC25A22	SLC25A22 (solute carrier family 25 member 22)	79751
SLC25A24	SLC25A24 (solute carrier family 25 member 24)	29957
SLC25A26	SLC25A26 (solute carrier family 25 member 26)	115286
SLC25A28	SLC25A28 (solute carrier family 25 member 28)	81894
SLC25A34	SLC25A34 (solute carrier family 25 member 34)	284723
SLC25A38	SLC25A38 (solute carrier family 25 member 38)	54977
SLC25A41	SLC25A41 (solute carrier family 25 member 41)	284427
SOX4	SOX4 (SRY-box 4)	6659
SRP14	SRP14 (signal recognition particle 14)	6727
SRP72	SRP72 (signal recognition particle 72)	6731
SRPRB	SRPRB (SRP receptor subunit beta)	58477
STAT3	STAT3 (signal transducer and activator of transcription 3)	6774
TARS2	TARS2 (threonyl-tRNA synthetase 2, mitochondrial)	80222
TSFM	TSFM (Ts translation elongation factor, mitochondrial)	10102
TUFM	TUFM (Tu translation elongation factor, mitochondrial)	7284
UCN	UCN (urocortin)	7349
UPF3A	UPF3A (UPF3A regulator of nonsense mediated mRNA decay)	65110
UQCC2	UQCC2 (ubiquinol-cytochrome c reductase complex assembly factor 2)	84300
VARS	VARS (valyl-tRNA synthetase)	7407
WARS2	WARS2 (tryptophanyl tRNA synthetase 2, mitochondrial)	10352
WIBG	PYM1 (PYM homolog 1, exon junction complex associated factor)	84305
XPO5	XPO5 (exportin 5)	57510
YARS	YARS (tyrosyl-tRNA synthetase)	8565
YBX2	YBX2 (Y-box binding protein 2)	51087

Supplemental Table S6 - 187 Gene Signature Functional Enrichments

GO: Cellular Component

ID	Name	Source	pValue	FDR B&H	Bonferroni	Genes from Input	Genes in Annotation
GO:0010494	cytoplasmic stress granule		6.85E-06	5.48E-05	2.19E-03	7	77

cytoplasmic stress granule; GO:0010494

Entrez Gene ID	Gene Symbol	Gene Name	Original Symbol
23367	LARP1	La ribonucleoprotein 1, translational regulator	*LARP1*
23369	PUM2	pumilio RNA binding family member 2	*PUM2*
26986	PABPC1	poly(A) binding protein cytoplasmic 1	*PABPC1*
1656	DDX6	DEAD-box helicase 6	*DDX6*
29118	DDX25	DEAD-box helicase 25	*DDX25*
1994	ELAVL1	ELAV like RNA binding protein 1	*ELAVL1*
4076	CAPRIN1	cell cycle associated protein 1	*CAPRIN1*

Pubmed

ID	Name	Source	pValue	FDR B&H	Bonferroni	Genes from Input	Genes in Annotation
29721183	Delineating the HMGB1 and HMGB2 interactome in prostate and ovary epithelial cells and its relationship with cancer.	Pubmed	2.02E-62	1.57E-59	5.19E-58	44	188

Entrez Gene ID	Gene Symbol	Gene Name	Original Symbol
6147	RPL23A	ribosomal protein L23a	RPL23A
4869	NPM1	nucleophosmin 1	NPM1
6152	RPL24	ribosomal protein L24	RPL24
6154	RPL26	ribosomal protein L26	RPL26
6155	RPL27	ribosomal protein L27	RPL27
6160	RPL31	ribosomal protein L31	RPL31
6161	RPL32	ribosomal protein L32	RPL32
25873	RPL36	ribosomal protein L36	RPL36
6175	RPLP0	ribosomal protein lateral stalk subunit P0	RPLP0
6187	RPS2	ribosomal protein S2	RPS2
26156	RSL1D1	ribosomal L1 domain containing 1	RSL1D1
6191	RPS4X	ribosomal protein S4 X-linked	RPS4X
6193	RPS5	ribosomal protein S5	RPS5
6194	RPS6	ribosomal protein S6	RPS6
6202	RPS8	ribosomal protein S8	RPS8
6203	RPS9	ribosomal protein S9	RPS9
6204	RPS10	ribosomal protein S10	RPS10
6206	RPS12	ribosomal protein S12	RPS12
6207	RPS13	ribosomal protein S13	RPS13
6209	RPS15	ribosomal protein S15	RPS15
6210	RPS15A	ribosomal protein S15a	RPS15A
23367	LARP1	La ribonucleoprotein 1, translational regulator	LARP1
60488	MRPS35	mitochondrial ribosomal protein S35	MRPS35
6218	RPS17	ribosomal protein S17	RPS17
6222	RPS18	ribosomal protein S18	RPS18
9045	RPL14	ribosomal protein L14	RPL14
6229	RPS24	ribosomal protein S24	RPS24
6234	RPS28	ribosomal protein S28	RPS28
6235	RPS29	ribosomal protein S29	RPS29
4736	RPL10A	ribosomal protein L10a	RPL10A
7818	DAP3	death associated protein 3	DAP3
64949	MRPS26	mitochondrial ribosomal protein S26	MRPS26
51649	MRPS23	mitochondrial ribosomal protein S23	MRPS23
708	C1QBP	complement C1q binding protein	C1QBP
65993	MRPS34	mitochondrial ribosomal protein S34	MRPS34
23521	RPL13A	ribosomal protein L13a	RPL13A
6122	RPL3	ribosomal protein L3	RPL3
6128	RPL6	ribosomal protein L6	RPL6
6130	RPL7A	ribosomal protein L7a	RPL7A
6132	RPL8	ribosomal protein L8	RPL8
6135	RPL11	ribosomal protein L11	RPL11
6136	RPL12	ribosomal protein L12	RPL12

6139	RPL17	ribosomal protein L17	RPL17
6141	RPL18	ribosomal protein L18	RPL18

Coexpression

ID	Name	Source	pValue	FDR B&H	Bonferroni	Genes from Input	Genes in Annotation
15529182-Table2	Human Prostate ChoVega05 44genes	GeneSigDB	5.46E-16	7.57E-14	3.79E-12	11	32

Human Prostate_ChoVega05_44genes

Entrez Gene ID	Gene Symbol	Gene Name	Original Symbol
6161	RPL32	ribosomal protein L32	RPL32
25873	RPL36	ribosomal protein L36	RPL36
6167	RPL37	ribosomal protein L37	RPL37
6171	RPL41	ribosomal protein L41	RPL41
6202	RPS8	ribosomal protein S8	RPS8
6222	RPS18	ribosomal protein S18	RPS18
6229	RPS24	ribosomal protein S24	RPS24
6235	RPS29	ribosomal protein S29	RPS29
9349	RPL23	ribosomal protein L23	RPL23
23521	RPL13A	ribosomal protein L13a	RPL13A
6139	RPL17	ribosomal protein L17	RPL17

Supplemental Table S7 - cBioPortal analysis of the 187 Gene Signature.

Table S7 provided as an excel file.

Supplemental Table S8

Univariate differences in patient characteristics as outlined below were evaluated using a 2-sample t-test or Wilcoxon rank sum test where appropriate.

ALL PATIENTS

Variable	African Americans (n = 33)	Caucasians (n = 26)	*P*
Age, years, mean (SD)	64.6 (6.6)	65.7 (6.5)	0.472
Blood Pressure, mmHg , mean (SD)	SBP: 136.5 (17.9)	SBP: 135.8 (17.9)	0.874
	DBP: 82.3 (8.4)	DBP: 80.7 (9.7)	0.487
Body Mass Index, kg/m2, mean (SD)	29.8 (7.1)	29.0 (3.8)	0.635
Vitamin D, IU, mean (SD)	26.3 (14.3)	34.9 (13.2)	0.021
Total cholesterol, mg/dL, mean (SD)	199.6 (40.3)	179.4 (39.7)	0.059
Hba1c, mmol/mol, median (IQR)	5.70 (0.70)	5.45 (0.70)	0.057
Prostate Specific Antigen, ng/mL, median (IQR)	6.12 (2.60)	6.47 (3.70)	0.676
DHEA-S, mg/dL, median (IQR)	121.0 (109.0)	114.5 (90.0)	0.676
C-reactive protein, mg/dL, median (IQR)	0.19 (0.30)	0.23 (0.43)	0.964

CANCER PATIENTS

Variable	African Americans (n = 27)	Caucasians (n = 12)	*P*
Age, years, mean (SD)	64.6 (6.7)	65.6 (7.7)	0.674
Blood Pressure, mmHg , mean (SD)	SBP: 138.2 (17.9)	SBP: 132.3 (15.5)	0.328
	DBP: 83.7 (7.1)	DBP: 82.1 (9.9)	0.565
Body Mass Index, kg/m2, mean (SD)	29.6 (7.2)	28.7 (4.2)	0.689
Vitamin D, IU, mean (SD)	25.2 (13.3)	38.0 (15.8)	0.013
Total cholesterol, mg/dL, mean (SD)	196.7 (41.9)	184.2 (46.6)	0.410
Hba1c, mmol/mol, median (IQR)	5.60 (0.80)	5.60 (0.85)	0.728
Prostate Specific Antigen, ng/mL, median (IQR)	6.21 (2.28)	7.61 (3.09)	0.443
Grade, median (IQR)	7 (1.5)	6 (7)	0.132
Number positive cores, median (IQR)	2 (3)	1 (1.5)	0.004
DHEA-S, mg/dL, median (IQR)	114.0 (119.0)	127.5 (67.5)	0.856
C-reactive protein, mg/dL, median (IQR)	0.19 (0.53)	0.24 (0.35)	0.577

BENIGN PATIENTS

Variable	African Americans (n = 6)	Caucasians (n = 14)	*P*
Age, years, mean (SD)	63.7 (6.3)	65.7 (6.2)	0.509
Blood Pressure, mmHg , mean (SD)	SBP: 129.0 (17.1)	SBP: 138.8 (19.9)	0.308
	DBP: 76.0 (11.2)	DBP: 79.4 (9.8)	0.501
Body Mass Index, kg/m2, mean (SD)	30.7 (7.6)	29.4 (3.6)	0.698
Vitamin D, IU, mean (SD)	31.3 (18.5)	32.3 (10.3)	0.873
Total cholesterol, mg/dL, mean (SD)	212.8 (32.4)	175.3 (34.0)	0.034
Hba1c, mmol/mol, median (IQR)	5.75 (0.70)	5.25 (0.50)	0.053
Prostate Specific Antigen, ng/mL, median (IQR)	5.2 (4.3)	5.8 (2.5)	0.544
DHEA-S, mg/dL, median (IQR)	172.0 (168.0)	109.0 (131.0)	0.443

C-reactive protein, mg/dL, median (IQR)	0.22 (0.22)	0.22 (0.31)	0.968

5 EXTREMES					
**Variable**	**N**	**Mean**	**Std Dev**	**Minimum**	**Maximum**
Systolic Blood pressure	5	138.2	26.61	120	183
Diastolic Blood Pressure	5	85.2	7.6	77	94
BMI	5	30.19	5.77	21.6	37.6
Vitamin D level	5	20.32	13.33	4.8	38
Total Cholesterol	5	176.2	56.49	85	225
HbA1c	5	5.92	2.35	4.1	10
Grade/Gleason Score	5	6.9	0.55	6	7.5
PSA	5	9.31	6.83	5.69	21.5
Number of Positive Cores	5	6.6	4.04	1	11
DHEAS	5	205.4	119.87	36	297
CRP	5	0.2	0.1	0.1	0.4

Supplemental Table S9
Clinical characteristics of the five extremal patients.

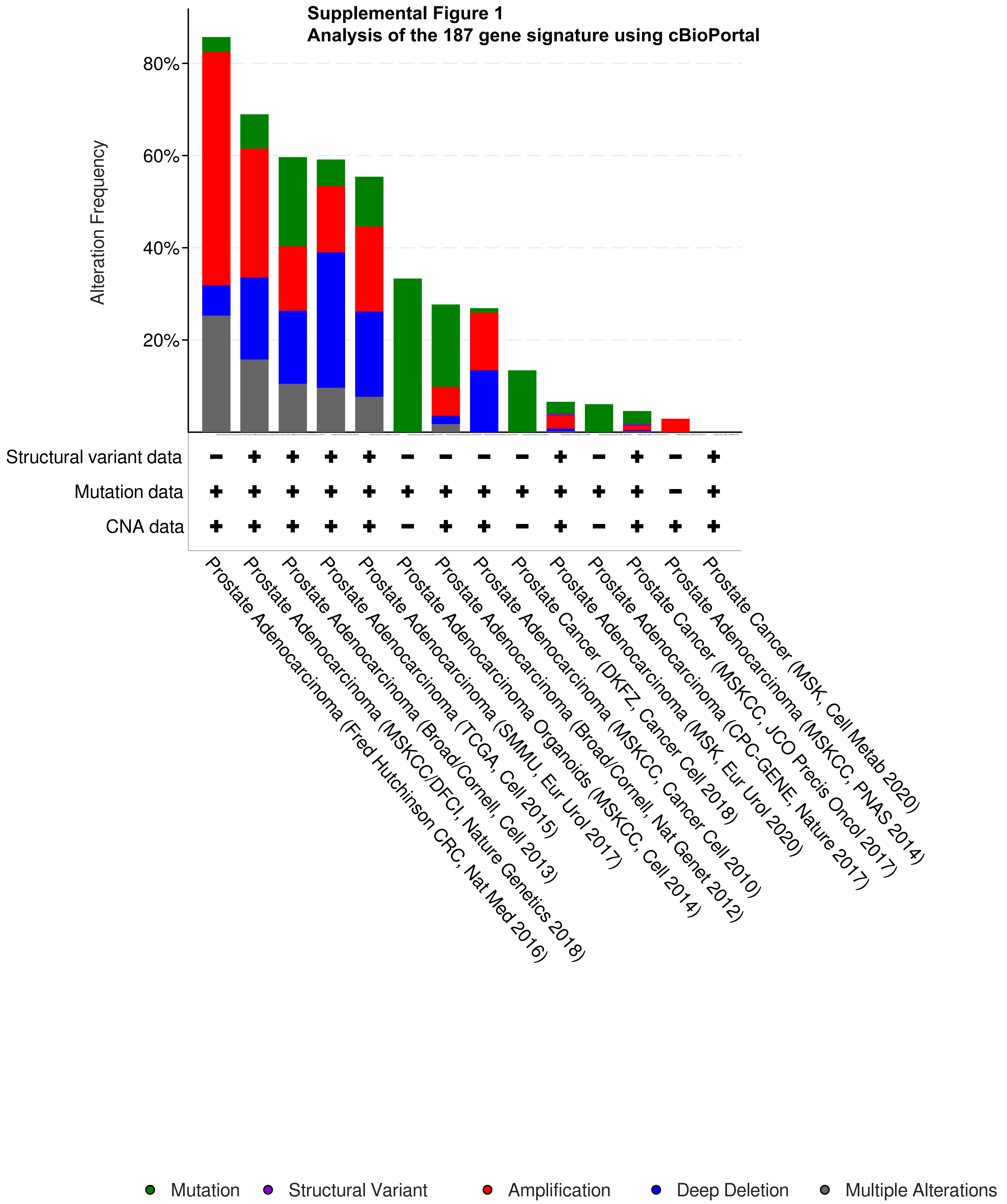
Supplemental Figure 1
Analysis of the 187 gene signature using cBioPortal
Alteration Frequency
80%
60%
40%
20%
Structural variant data
Mutation data
CNA data
Prostate Adenocarcinoma (Fred Hutchinson CRC, Nat Med 2016)
Prostate Adenocarcinoma (MSKCC/DFCI, Nature Genetics 2018)
Prostate Adenocarcinoma (Broad/Cornell, Cell 2013)
Prostate Adenocarcinoma (TCGA, Cell 2015)
Prostate Adenocarcinoma (SMMU, Eur Urol 2017)
Prostate Adenocarcinoma Organoids (MSKCC, Cell 2014)
Prostate Adenocarcinoma (Broad/Cornell, Nat Genet 2012)
Prostate Adenocarcinoma (MSKCC, Cancer Cell 2010)
Prostate Cancer (DKFZ, Cancer Cell 2018)
Prostate Adenocarcinoma (MSK, Eur Urol 2020)
Prostate Adenocarcinoma (CPC-GENE, Nature 2017)
Prostate Cancer (MSKCC, JCO Precis Oncol 2017)
Prostate Adenocarcinoma (MSKCC, PNAS 2014)
Prostate Cancer (MSK, Cell Metab 2020)
Mutation
Structural Variant
Amplification
Deep Deletion
Multiple Alterations
